# Genetic characterization and evidence for multiple reassortments of rotavirus A G3P[3] in dogs and cats in Thailand

**DOI:** 10.3389/fvets.2024.1415771

**Published:** 2024-05-24

**Authors:** Ekkapat Chamsai, Kamonpan Charoenkul, Kitikhun Udom, Waleemas Jairak, Supassama Chaiyawong, Alongkorn Amonsin

**Affiliations:** ^1^Center of Excellence for Emerging and Re-emerging Infectious Diseases in Animals, and One Health Research Cluster, Faculty of Veterinary Science, Chulalongkorn University, Bangkok, Thailand; ^2^Department of Veterinary Public Health, Faculty of Veterinary Science, Chulalongkorn University, Bangkok, Thailand

**Keywords:** cats, dogs, genetic characterization, interspecies transmission, reassortment, rotavirus A

## Abstract

Rotavirus A (RVA) causes gastroenteritis in humans and animals. The zoonotic potential of RVA has been reported and raises major concerns, especially in animal-human interface settings. The study aimed to characterize and investigate the genetic diversity among RVAs in dogs and cats in Thailand. We collected 572 rectal swab samples from dogs and cats in Bangkok animal hospitals from January 2020 to June 2021. The one-step RT-PCR assay detected RVAs in 1.92% (11/572) of the samples, with 2.75% (8/290) in dogs and 1.06% (3/282) in cats. Two canine RVA and one feline RVA were subjected to whole genome sequencing. Our results showed that all three viruses were identified as RVA genotype G3P[3]. The genetic constellation of RVAs is unique for different species. For canine RVAs is G3-P [3]-I3-R3-C3-M3-A9-N2-T3-E3-H6, while Feline RVA is G3-P [3]-I8-R3-C3-M3-A9-N3-T3-E3-H6. Notably, both canine and feline RVAs contained the AU-1 genetic constellation with multiple reassortments. The results of phylogenetic, genetic, and bootscan analyses showed that canine RVAs may have reassorted from dog, human, and cat RVAs. While feline RVA was closely related to RVAs in humans, bats, and simians. This study provided genetic characteristics and diversity of RVAs in dogs and cats and suggested possible multiple reassortments, suggesting the zoonotic potential of the viruses. Thus, public health awareness should be raised regarding the zoonotic potential of RVAs in dogs and cats. Further studies on RVAs on a larger scale in dogs and cats in Thailand are needed.

## Introduction

Rotavirus (RV) is a non-enveloped, double-stranded RNA virus of the family *Sedoreoviridae*, genus *Rotavirus*. The virus consists of 11 RNA segments encoding 6 structural proteins (VP1-VP4, VP6, VP7) and 5 or 6 nonstructural proteins (NSP1-NSP5/NSP6). RV can be classified into species A to D and F to J (1). Among these, rotavirus A (RVA) is the most common rotavirus species causing gastroenteritis in animals and humans worldwide (2, 3). In humans, RVA infection causes severe diarrhea in children and can be fatal. Previous studies reported that 30–50% of children with diarrhea were infected with RVA (4, 5). Despite the use of the rotavirus vaccine, there are still gastroenteritis outbreaks from rotavirus worldwide, mainly in developing countries. The causes of RVA outbreaks could be due to the emergence of novel RVA strains or the reassortment of the RVAs from different origins. Some strains of RVAs have also been reported as having zoonotic potential. For instance, RVAs genotype G3[P3], G3[P9] from dogs, G9[P11], G10[P11] from cattle, and G4[P6] from pigs have been reported in Human (6–8). In animals, RVA infection causes gastroenteritis in several animal species. RVA infection affects animal production, especially in the cattle, swine, and poultry industries (9, 10).

In domestic dogs and cats, RVA can cause mild diarrhea in puppies and kittens but it is mostly subclinical and self-limiting (11). G3[P3] is the predominant RVA genotype in dogs, while genotype G3[P9] is more frequently infected in cats (12, 13). The evidence of interspecies transmission of RVA from dogs and cats to humans, as well as the reassortment among RVAs, have been reported (8, 9, 14–16). For example, a unique G3[P3] was detected in Brazil and demonstrated the possible canine-to-human transmission (14). Some previous studies reported interspecies transmission of RVAs between dogs and children with severe diarrhea (15, 17). Evidence of multiple reassortments of RVAs between feline and human rotaviruses has also been documented (8). Since dogs and cats usually interface with humans, rotavirus can be transmitted by the fecal-oral route. Thus, RVAs can spread easily and increase the chance of interspecies transmission or viral reassortment, which subsequently generates novel strains of RVAs with zoonotic potential. The objective of this study was to determine genetic characteristics and investigate the genetic diversity of RVA circulating in domestic dogs and cats in Thailand.

## Materials and methods

### Sample collection from domestic dogs and cats

A total of 572 rectal swab samples were collected from domestic dogs (*n* = 290) and cats (*n* = 282) from January 2020 to June 2021. The samples were collected from animals of all ages, sexes, breeds, and health conditions during the visit to various animal hospitals (*n* = 8) in Bangkok and Nonthaburi, Thailand. The animal’s demographic data, including age, sex, and health condition, were also recorded. The rectal swabs were collected and placed in viral transport media (MEM; eagle minimum essential medium), kept at 4°C, and transported to the laboratory within 24 h. This study was conducted under the approval of the Faculty of Veterinary Science, Chulalongkorn University’s Animal Use and Care Committee (IACUC #2031035 and #2031050).

### Identification of RVA in domestic dogs and cats by specific RT-PCR assay

RNA extraction was performed by using GeneAll^®^ GENTiTM Viral DNA/RNA Extraction Kit (GeneAll^®^; Lisbon, Portugal) on a GENTiTM 32 (GeneAll^®^; Lisbon, Portugal) following the manufacturer’s instructions. In brief, 200 μL of the supernatant from rectal swab samples were mixed with lysis buffer and 7 μL of RNA carrier in the GeneAll^®^ GENTiTM Viral DNA/RNA Extraction Kit (GeneAll^®^; Lisbon, Portugal). The kit was processed using a GENTiTM 32 extraction machine (GeneAll^®^; Lisbon, Portugal) to extract RNA. The extracted RNA was eluted by the elution buffer and stored at −20°C until virus identification.

The RNA samples were subjected to rotavirus A identification by one-step RT-PCR using primers specific to the VP6 and NSP5 genes of RVA as previously described with some modifications (18). The one-step RT-PCR for rotavirus A identification was performed by using SuperScript^™^ III One-Step RT-PCR System with Platinum^™^ Taq DNA Polymerase (Invitrogen^™^). In brief, a 25 μL one-step RT-PCR reaction contained 3 μL template RNA, 12.5 μL 2xReaction Mix, 0.5 μL 10 μM forward and reverse primers, 1 μL SuperScript III RT (Invitrogen, CA), and distilled water. The RT-PCR assay conditions were: a cDNA synthesis step at 55°C for 30 min, an initial denaturation step at 94°C for 2 min, followed by 40 cycles of denaturation at 94°C for 30 s, annealing at 52°C for 45 s, extension at 68°C for 1 min, and a final extension step at 68°C for 5 min.

### Genetic characterization of RVA in dogs and cats by whole genome sequencing

The RVAs from dogs and cats were subjected to whole-genome sequencing. Each gene of RVA (VP1, VP2, VP3, VP4, VP6, VP7, NSP1, NSP2, NSP3, NSP4, NSP5) was amplified by using primers previously described (Supplementary Table S1). In brief, one-step RT-PCR was conducted by using SuperScript^™^ III One-Step RT-PCR System with Platinum^™^ Taq DNA Polymerase (Invitrogen^™^) in a final volume of 25 μL comprised of 3 μL of template RNA, 12.5 μL of 2xReaction Mix, 0.5 μL of 10 μM forward and reverse primers, 1 μL of SuperScript III RT (Invitrogen, CA) and distilled water to a final volume. The RT-PCR assay included a cDNA synthesis step at 55°C for 30 min, an initial denaturation step at 94°C for 2 min, followed by 40 cycles of denaturation at 94°C for 30 s, annealing at 45–53°C for 45 s, and extension at 68°C for 1–4 min, as well as a final extension step at 68°C for 5 min. Agarose gel electrophoresis was performed to confirm positive PCR amplification. The expected PCR product size is provided in Supplementary Table S1.

In this study, three RVAs (CU25012, CU25045, and CU25170) with high RNA quality were subjected to whole genome sequencing. In addition, two RVAs (CU24998 and CU25171) were subjected to VP7 and VP4 amplification and sequencing. The selection criteria for RVA representative samples were based on the host species of RVAs, time of collection, and quality of RNA. PCR products of each gene were then pooled together and purified by NucleoSpin^®^ Gel and PCR Clean-up (MACHEREY-NAGEL^™^, Germany). The Oxford Nanopore sequencing technology was used for whole genome sequencing. While VP4 and VP7 sequencing, Sanger’s sequencing was carried out. First, the purified PCR products were subjected to sequencing by Oxford Nanopore sequencing device and Minion flow cells with Rapid sequencing kit (Cat#SQK-RAD004) (ONT, UK). The DNA library and the flow cells priming mix were prepared following the manufacturer’s instructions. In detail, first, the number of pores in the flow cell was checked via the MinKNOW software to ensure the ability of sequencing. The number of pores should not be lower than 200 to ensure the quality of sequences. To prepare the DNA library, 7.5 μL of PCR product was mixed with 2.5 μL of fragmentation mix (FRA) and incubated at 30°C for 1 min, then at 80°C for 1 min. After cooling on ice, 1 μL of rapid adapter (RAP) was added and incubated at room temperature for 5 min. Finally, 34 μL of sequencing buffer (SQB), 25.5 μL of loading beads (LB), and 4.5 μL of nuclease-free water were added to complete the DNA library preparation. The flow cell priming mix was prepared by mixing 30 μL of flush tether (FLT) with flush buffer (FB). To start the sequencing process, 800 μL of flow cell priming mix was added to the flow cell and incubated for 5 min. Next, another 200 μL of flow cell priming mix was added, followed by 75 μL of prepared DNA library. Finally, the sequencing process was initiated through the MinKNOW software.

The MinKNOW software was used for sequence reading and base-calling processes to convert the electrical signal (fast5 file) to nucleotide (fastq file). The minimum Qscore 7 was used to remove the poor-quality sequence from the base-calling process. The nucleotide sequences were assembled through de-novo assembly using the Genome Detective web software.^1^ The de-novo assembly process was performed using mapped to reference RVAs in the web database. The nucleotide sequence of each gene segment of RVAs was retrieved in fasta file format and compared to the NCBI database using BLAST to identify the closest matching reference sequence for each gene segment. The original fastq file from MinKNOW software was then mapped to the reference sequences using CLC Genomic Workbench software 20.0 (Qiagen, Hilden, Germany). Finally, the consensus sequence of each virus gene segment was exported in fasta file format for analysis.

### Genotyping and genetic diversity of RVA in dogs and cats

To identify the genotype of RVA, the nucleotide sequences of all 11 segments were amplified using primers previously described (Supplementary Table S1). Then, purified PCR products were subjected to sequencing using Oxford Nanopore sequencing technology. The nucleotide sequences of all gene segments were analyzed using the Virus Pathogen Resource (ViPR) web software.^2^ The algorithm used to identify the genotype of RVA is based on the RotaC algorithm (19). Each gene segment’s fasta file format was uploaded into the ViPR web program to assign the genotype. The genetic constellation of RVA was identified by using the combination of 11 genotypes from each gene segment. The genetic constellation of RVAs in this study was then compared with RVAs from various species, such as dogs, cats, pigs, horses, bats, and humans.

The nucleotide and amino acid identities of each gene segment from RVAs in the study were compared to reference RVAs of the same genotype by using a pairwise distance function in MEGA v10.0 software. The nucleotide sequences of RVAs in this study, as well as the reference RVAs, were aligned using the ClustalW function. Only the coding region of each segment of the RVA was used to calculate the percentage of similarity of each segment of the virus using MEGA software. The phylogenetic tree of each segment (11 segments) was generated by using MEGA v10. 0 software with a maximum-likelihood method with 1,000 bootstrap replicates. The best substitution model for tree construction for each gene was based on the lowest Bayesian information criterion, with JTT for VP7/NSP2/NSP4/NSP5, JTT + F for VP4/VP3/NSP1, and LG for VP6/VP1/VP2/NSP3 (20). The nucleotide sequences of reference RVAs from dogs, cats, humans, pigs, horses, and bats were also included in phylogenetic analysis.

Reassortment analysis of RVA was performed by using vistaplot and bootscan analysis. The vistaplot analysis was generated by wgVISTA web software to compare the RVAs in this study with closely related strains (21). In brief, the nucleotide sequences of the coding region from all 11 RVA segments were concatenated. The concatenated sequence was then uploaded to the wgVISTA web software along with the closely related strains to compare the similarity between the concatenated sequences. For bootscan analysis, the SimPlot v.3.5.1 program was used in this study. In brief, the concatenated nucleotide sequence of RVAs in this study and closely related strains were deposited in the SimPlot v.3.5.1 program and analyzed by bootscan function with a neighbor-joining model, 1,000 bootstrap replicates, Kimura (2-parameter) distance model, 1,000 bp window size, and 70 bp step size.

## Result

In this study, we tested 572 rectal swab samples from dogs and cats with RVA-specific primers and found that 11 (1.92%) were positive for RVA. In detail, 8 out of 290 (2.75%) rectal swab samples from dogs were positive for canine rotavirus A, and 3 out of 282 (1.06%) rectal swab samples from cats were positive for feline rotavirus A. By age of animals, there were 5 dogs under 6 months old, 3 dogs over 10 years old, 2 cats were 1 and 3 years old, and 1 cat under 6 months old. By sex of animals, out of 11 animals, 6 dogs were male, and 2 were female, while all 3 cats were male. All animals were clinically healthy during sample collection. It is noted that most positive samples were collected from animal hospitals in Bangkok, except only 1 dog sample was collected from animal hospitals in Nonthaburi province.

In this study, three RVAs (CU25012, CU25045, and CU25170) were subjected to whole genome sequencing. In addition, two RVAs (CU24998 and CU25171) were subjected to VP7 and VP4 sequencing. Whole genome sequencing of RVAs from dogs (CU25012 and CU25170) and cats (CU25045) was accomplished in all samples except the partial VP1 gene of CU25170. Two canine RVAs (CU25012 and CU25170) were recovered from dogs in January 2020 and July 2020, while one feline RVA (CU25045) was collected from a cat in February 2020. The whole genome and gene sequences of RVAs were deposited in the GenBank database (Table 1).

**Table 1:** Description of canine and feline rotaviruses characterized in this study.

ID	Date	Species	Age	Sex	Province	Genotype	Sequencing	GenBank accession #
Feline Rotavirus								
CU25045	Feb-20	Cat	3 mths	Male	Bangkok	FRV-G3-P[3]	WGS*	OR501082-92
Canine Rotavirus								
CU25012	Jan-20	Dog	3 mths	Male	Bangkok	CRV-G3-P[3]	WGS*	OR501093-103
CU25170	Jul-20	Dog	3 mths	Male	Bangkok	CRV-G3-P[3]	WGS*	OR501104-14
CU24998	Jan-20	Dog	2 mths	Male	Bangkok	CRV-G3-P[3]	VP7, VP4**	OR501117-18
CU25171	Jul-20	Dog	3 mths	Male	Bangkok	CRV-G3-P[3]	VP7, VP4**	OR501115-16

*WGS, whole genome sequences (11 gene segments) and **VP7, VP4, VP7 and VP4 sequences are available for genotyping.

Genotyping of RVAs by using Virus pathogen resource (ViPR) showed that three whole genome sequences (*n* = 3) and VP7 and VP4 sequences (*n* = 2) of Thai-Canine RVAs and Feline RVA belong to RVA genotype G3P[3]. Analysis of the whole genome sequences showed that canine RVAs (CU25012, CU25170) were assigned to genotype G3-P[3]-I3-R3-C3-M3-A9-N2-T3-E3-H6, which is identical to the genotype of Canine RVA previously reported in Thailand (18). On the other hand, the feline RVA (CU25045) was classified as genotype G3-P[3]-I8-R3-C3-M3-A9-N3-T3-E3-H6, which has never been reported in domestic cats in Thailand. Additionally, the genotype I8 of the VP6 gene of feline RVAs has never been reported in cats in the GenBank database (Table 2).

**Table 2:** Genetic constellation of canine RVAs and feline RVAs and reference RVAs from dogs, cats, horses, bats, ruminants, avians, pigs, and humans.

Virus	Strain	Species	Year	Country	Gene										
					VP7	VP4	VP6	VP1	VP2	VP3	NSP1	NSP2	NSP3	NSP4	NSP5
This study															
Canine															
RVA/Dog/THA/CU25012/2020/G3P[3]*	CU25012	Dog	2020	THA	G3	P[3]	I3	R3	C3	M3	A9	N2	T3	E3	H6
RVA/Dog/THA/CU25170/2020/G3P[3]*	CU25170	Dog	2020	THA	G3	P[3]	I3	X	C3	M3	A9	N2	T3	E3	H6
Feline															
RVA/Cat/THA/CU25045/2020/G3P[3]*	CU25045	Cat	2020	THA	G3	P[3]	I8	R3	C3	M3	A9	N3	T3	E3	H6
Reference strain															
Canine															
RVA/Dog-tc/USA/A79-10/1979/G3P[3]	A79-10	Dog	1979	USA	G3	P[3]	I3	R3	C2	M3	A9	N2	T3	E3	H6
RVA/Dog-tc/USA/K9/1981/G3P[3]	K9	Dog	1981	USA	G3	P[3]	I3	R3	C2	M3	A9	N2	T3	E3	H6
RVA/Dog-tc/USA/CU-1/1982/G3P[3]	CU-1	Dog	1982	USA	G3	P[3]	I3	R3	C2	M3	A9	N2	T3	E3	H6
RVA/Dog-tc/JPN/RS15/1982/G3P[3]	RS15	Dog	1982	JPN	G3	P[3]	I3	R3	C2	M3	A9	N3	T3	E3	H6
RVA/Dog-tc/ITA/RV198-95/1995/G3P3	RV198-95	Dog	1995	ITA	G3	P[3]	I3	R3	C2	M3	A9	N2	T3	E3	H6
RVA/Dog-tc/ITA/RV52-96/1996/G3P[3]	RV52-96	Dog	1996	ITA	G3	P[3]	I3	R3	C2	M3	A9	N2	T3	E3	H6
RVA/Dog/THA/CU132/2017/G3P[3]**	CU132	Dog	2017	THA	G3	P[3]	I3	R3	C3	M3	A9	N2	T3	E3	H6
RVA/Dog/THA/CU20139/2017/G3P[3]**	CU20139	Dog	2017	THA	G3	P[3]	I3	R3	C3	M3	A9	N2	T3	E3	H6
RVA/Dog/THA/CU23379/2019/G3P[3]**	CU23379	Dog	2019	THA	G3	P[3]	I3	R3	C3	M3	A9	N2	T3	E3	H6
Feline															
RVA/Cat-tc/AUS/Cat97/1984/G3P[3]	Cat97	Cat	1984	AUS	G3	P[3]	I3	R3	C2	M3	A9	N2	T3	E3	H6
RVA/Cat-tc/AUS/Cat2/1984/G3P[9]	Cat2	Cat	1984	AUS	G3	P[9]	I3	R3	C3	M3	A3	N1	T6	E3	H3
RVA/Cat/JPN/FRV348/1994//G3P[3]	FRV348	Cat	1994	JPN	G3	P[3]	I3	R3	C3	M3	A15	N3	T3	E3	H6
RVA/Cat/JPN/FRV384/1994//G3P[3]	FRV384	Cat	1994	JPN	G3	P[9]	I3	R3	C3	M3	A3	N3	T3	E3	H3
RVA/Cat-wt/ITA/BA222/2005/G3P[9]	BA222	Cat	2005	ITA	G3	P[9]	I2	R2	C2	M2	A3	N1	T3	E2	H3
Equine															
RVA/Horse-wt/ARG/E30/1993/G3P[12]	E30	Horse	1993	ARG	G3	P[12]	I6	R2	C2	M3	A10	N2	T3	E12	H7
RVA/Horse/ARG/E3198/2008/G3P[3]	E3198	Horse	2008	ARG	G3	P[3]	I3	R3	C3	M3	A9	N3	T3	E3	H6
Bat															
RVA/Bat-wt/ZMB/LUS12-14/2012/G3P[3]	LUS12-14	Bat	2012	ZMB	G3	P[3]	I3	R2	C2	M3	A9	N2	T3	E2	H3
RVA/Bat-wt/CHN/MSLH14/2012/G3P[3]***	MSLH14	Bat	2012	CHN	G3	P[3]	I8	R3	C3	M3	A9	N3	T3	E3	H6
RVA/Bat-wt/CHN/MYAS33/2012/G3P[10]	MYAS33	Bat	2013	CHN	G3	P[10]	I8	R3	C3	M3	A9	N3	T3	E3	H6
RVA/Bat-wt/CMR/BatLi09/2014/G30P[42]	BatLi09	Bat	2014	CMR	G30	P[42]	I22	R15	C15	M14	A25	N15	T17	E22	H17
RVA/Bat-wt/CHN/LZHP2/2015/G3P[3]	LZHP2	Bat	2015	CHN	G3	P[3]	I3	R3	C3	M3	A9	N3	T3	E3	H6
Ruminant															
RVA/Cow-tc/USA/WC3/1981/G6P[5]	WC3	Bovine	1981	USA	G6	P[5]	I2	R2	C2	M2	A3	N2	T6	E2	H3
Avian															
RVA/Avian/JPN/PO13/1988/G18P[17]	PO13	Avian	1988	JPN	G18	P[17]	I4	R4	C4	N4	A4	N4	T4	E4	H4
Swine															
RVA/Pig-tc/MEX/YM/1983/G11P[7]	YM	Porcine	1983	MEX	G11	P[7]	I5	R1	C1	M1	A8	N1	T1	E1	H1
RVA/Pig-tc/VEN/A131/1988/G3P[7]	A131	Porcine	1988	VEN	G3	P[7]	I5	R1	C2	M1	A1	N1	T1	E1	H1
RVA/Pig-tc/VEN/A253/1988/G11P[7]	A253	Porcine	1988	VEN	G11	P[7]	I5	R1	C2	M1	A1	N1	T1	E1	H1
RVA/Pig-tc/ESP/OSU-C5111/2010/GP[7]	OSU-C511	Porcine	2010	ESP	G5	P[7]	I5	R1	C1	M1	A1	N1	T1	E1	H1
Human															
RVA/Human-tc/USA/Wa/1974/G1P[8]	Wa	Human	1974	USA	G1	P[8]	I1	R1	C1	M1	A1	N1	T1	E1	H1
RVA/Human-tc/USA/DS-1/1976/G2P[4]	DS-1	Human	1976	USA	G2	P[4]	I2	R2	C2	M2	A2	N2	T2	E2	H2
RVA/Human-tc/JPN/AU-1/1982/G3P[9]	AU-1	Human	1982	JPN	G3	P[9]	I3	R3	C3	M3	A3	N3	T3	E3	H3
RVA/Human-tc/USA/HCR3A/1984/G3P[3]	HCR3A	Human	1984	USA	G3	P[3]	I3	R3	C2	M3	A9	N2	T3	E3	H6
RVA/Human-tc/ISR/Ro1845/1985/G3P[3]	Ro1845	Human	1985	ISR	G3	P[3]	I3	R3	C2	M3	A9	N2	T3	E3	H6
RVA/Human-wt/ITA/PAH136/1996/G3P[9]	PAH136	Human	1996	ITA	G3	P[9]	I2	R2	C2	M2	A3	N1	T6	E2	H3
RVA/Human-wt/ITA/PA158/1996/G3P[9]	PA158	Human	1996	ITA	G3	P[9]	I2	R2	C2	M2	A3	N2	T6	E2	H3
RVA/Human-tc/ITA/PA260-97/1997/G3P[3]	PA260-97	Human	1997	ITA	G3	P[3]	I3	R3	C3	M3	A15	N2	T3	E3	H6
RVA/Human-tc/THA/T152/1998/G12P[9]	T152	Human	1998	THA	G12	P[9]	I3	R3	C3	M3	A12	N3	T3	E3	H6
RVA/Human-wt/THA/CMH222/2001/G3P[3]	CMH222	Human	2001	THA	G3	P[3]	I8	-	-	-	-	-	-	E3	-
RVA/Human-wt/THA/CMH079/2005/G3P[10]	CMH079	Human	2005	THA	G3	P[10]	I8	-	-	-	-	-	-	E3	H6
RVA/Human-tc/CHN/L621/2006/G3P[9]	L621	Human	2006	CHN	G3	P[9]	I3	R3	C3	M3	A3	N3	T3	E3	H6
RVA/Human-tc/THA/CU-365/2008/G3P[9]	CU365	Human	2008	THA	G3	P[9]	I3	R3	C3	M3	A3	N3	T3	E3	H6
RVA/Human/BRA/R2638/2011/G3P[3]	R2638	Human	2011	BRA	G3	P[3]	-	-	-	-	-	-	-	-	-
RVA/Human-wt/BRA/1A3739/2011/G3P9	1A3739	Human	2011	BRA	G3	P[9]	I18	R3	C3	Mx	A19	N3	T3	E3	H6
RVA/Human-wt/CHN/E2451/2011/G3P[9]	E2451	Human	2011	CHN	G3	P[9]	I3	R3	C3	M3	A3	N3	T3	E3	H6
RVA/Human-wt/JPN/12638/2014/G3P[3]**	12,638	Human	2014	JPN	G3	P[3]	I3	R3	C3	M3	A9	N2	T3	E3	H6
RVA/Human-wt/THA/MS2015-1-0001/2015/G3P[10]	MS2015-1-0001	Human	2015	THA	G3	P[10]	I8	R3	C3	M3	A9	N3	T3	E3	H6

*Canine and Feline RVA strain in this study, **Reference RVA strains with the same genetic constellation of Thai-Canine RVA, and ***Reference RVV strain with the same genetic constellation of Thai-Feline RVA.

**Figure 1:**
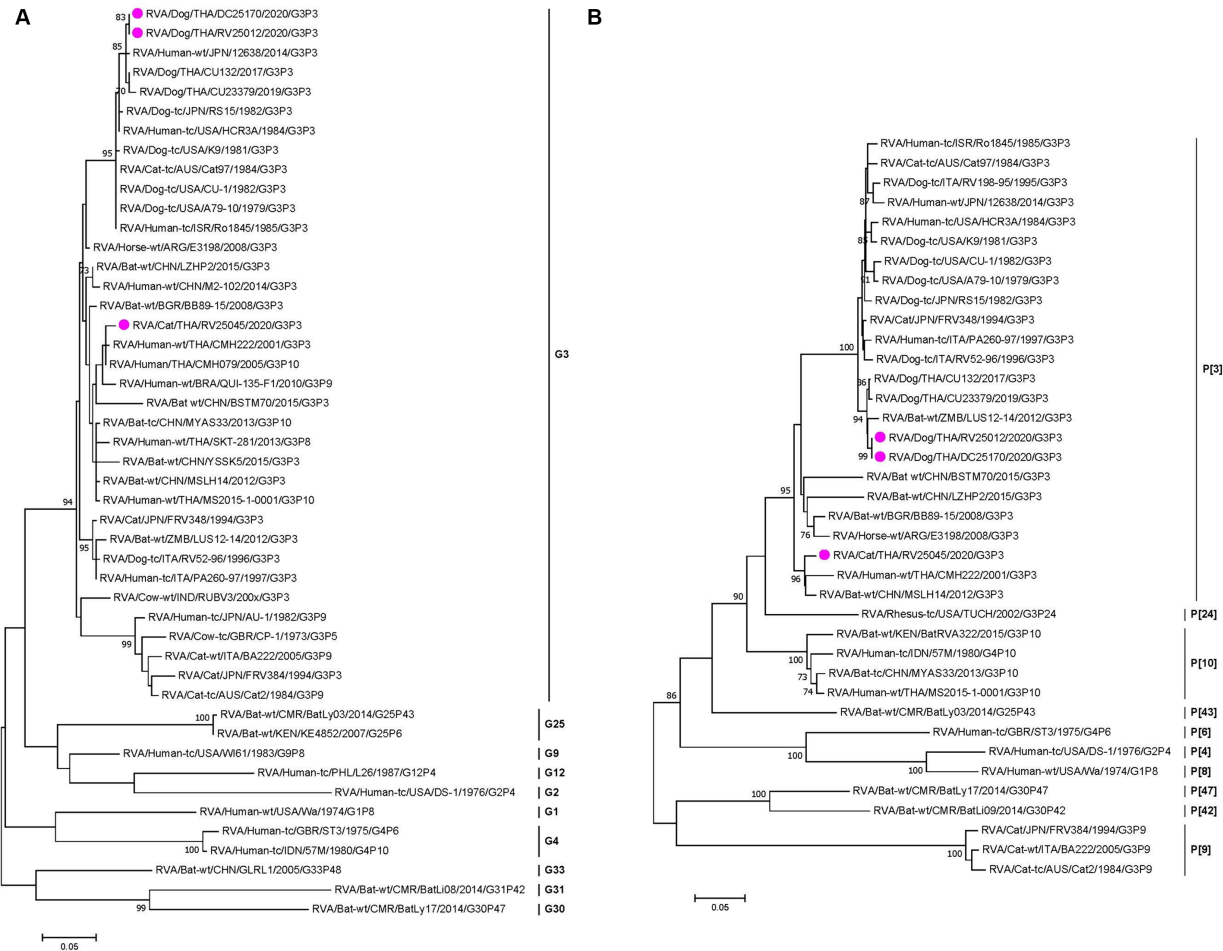
**(A)** Maximum-likelihood phylogenetic analysis of VP7 genes of canine RVAs and feline RVAs characterized in this study (indicated by the pink circle). The nucleotide sequences used in the analysis were 981 base pairs. Bootstrap values >70% are indicated at the tree nodes. Scale bars represent substitutions per nucleotide. **(B)** Maximum-likelihood Phylogenetic analysis of VP4 genes of canine RVAs and feline RVAs characterized in this study (indicated by the pink circle). The nucleotide sequences used in the analysis were 2,332 base pairs. Bootstrap values >70% are indicated at the tree nodes. Scale bars represent substitutions per nucleotide.

Phylogenetic analysis and pairwise comparison showed that two Thai-canine RVAs (CU25012 and CU25170) were closely related to previously reported canine RVAs in Thailand at 9 gene segments (VP1, VP2, VP3, VP4, VP7, NSP1, NSP2, NSP4, NSP5). However, the VP6 gene was more similar to human RVA from Japan (12638), and the NSP3 gene was closely related to feline RVA from Japan (FRV348) (18, 22, 23) (Figure 1; Supplementary Figure S1 and Supplementary Table S2). Phylogenetic analysis and pairwise comparison revealed that Thai-feline RVA (CU25045) was closely related to RVAs found in humans, bats, and simians. In detail, the VP7 and VP4 genes were closely related to human RVA (CMH222) in Thailand in 2001, and VP6 genes were closely related to human RVA (CMH079) in Thailand in 2005 (24, 25). In addition, VP3, NSP2, and NSP4 genes were also closely related to human RVA (MS2015-1-0001) in Thailand (26). Interestingly, some genes of Thai-feline RVA had high nucleotide similarities to bat RVA from China, MYAS33 (VP1, NSP1, and NSP3), MSLH14 (VP6, VP3, and NSP4), LZHP2 (VP4, NSP3, and NSP4 genes), and BSTM70 (VP6) (27, 28). Additionally, the VP2 gene of Thai feline RVA (CU25045) was closely related to simian RVA from the USA (29) (Figure 1; Supplementary Figure S2 and Supplementary Table S3).

The genetic constellation of the RVAs in this study showed that two canine RVAs (CU25012 and CU25170) are G3-P[3]-I3-R3-C3-M3-A9-N2-T3-E3-H6 genotype. Our result indicated an identical genetic constellation among canine RVAs from this study and canine RVAs from previous reports in Thailand (18). This unique genetic constellation has never been reported in dogs in other countries. Interestingly, there was a report of one human RVA (12638) from Japan that had the same genetic constellation (23). The genetic constellation of the feline RVA (CU25045) is G3-P[3]-I8-R3-C3-M3-A9-N3-T3-E3-H6 genotype. To the best of our knowledge, this genetic constellation of RVA has never been reported in any feline RVAs in the database. However, this genetic constellation had been found in bat RVA (MSLH14) reported in China (27) (Table 2).

**Figure 2:**
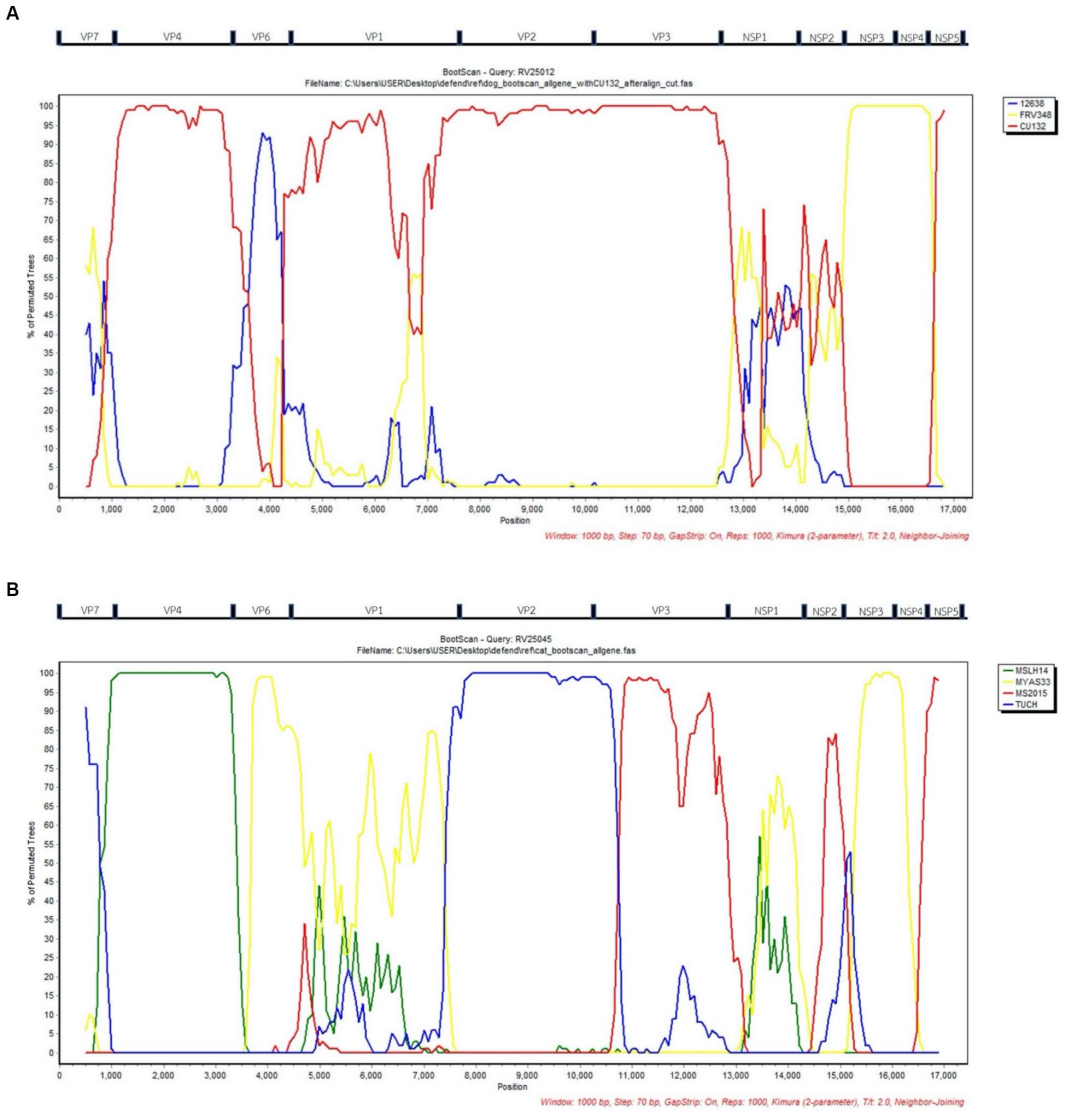
**(A)** Bootscan analysis of Canine RVA (CU25012) and reference RVAs from dog (CU132), human (12638), and cat (FRV348). Red line; Dog (CU132), Blue line; Human; (12638), Yellow line; Cat (FRV348). **(B)** Bootscan analysis of Feline RVA (CU25045) and reference RVAs from humans (MS2015-1-0001), bats (MYAS33 and MSLH14), and simians (TUCH). Red line; Human (MS2015-1-0001), Blue line; Simian (TUCH), Yellow line; Bat (MYAS33), Green line; Bat (MSLH14). *X*-axis; Nucleotide position, *Y*-axis; Percentage of permuted trees.

Bootscan analysis was conducted to analyze the reassortment event of the RVAs. For canine RVA (CU25012), the analysis showed the possibility of reassortment events of canine RVA in this study from human, dog, and cat RVAs (canine RVA (CU132); human RVA (12638); and feline RVA (FRV348)). In detail, VP4, VP2, VP3, NSP5 gene segments were from canine RVA (CU132), VP6 gene was from human RVA (12638) and NSP3, NSP4 gene segments were from feline RVA (FRV348). It is noted that VP7, VP1, NSP1, and NSP2 genes showed an inconclusive pattern, which might result from the reassortment of unknown RVAs (Figure 2A). Bootscan analysis of feline RVA (CU25045) showed the possibility of reassortment events from 4 closely related RVAs from humans, bats, and simians; bat RVA (MSLH14, MYAS33), human RVA (MS2015-1-0001) and simian RVA (TUCH). In detail, the VP4 gene was from bat RVA (MSLH14), and the VP6 and NSP3 genes were from bat RVA (MYAS33). While the VP3 and NSP4 genes were from human RVA (MS2015-1-0001), and the VP2 gene was from simian RVA (strain TUCH) (29). It is noted that VP1, VP7, NSP1, NSP2, and NSP5 genes showed inconclusive patterns (Figure 2B).

## Discussion

In this study, the occurrence of rotavirus A (RVA) from domestic dogs and cats was 1.92% (11/572). By species, the occurrence of canine RVA in domestic dogs was 2.75% (8/290), which was higher than that of 0.7% reported in a previous study in Thailand (18). However, the occurrence of canine RVA in this study was lower than in other countries such as Germany (39.7%), Iran (16.3%), Mexico (40%), and Brazil (8.2%) (9, 30–32). The occurrence of feline RVA in domestic cats was only 1.06% (3/282), which was lower than those reported in other studies, such as Germany (50.0%) and the UK (3.0%) (9, 33). In this study, 62.5% (5/8) of dogs with positive RVA were aged less than 6 months, which was similar to other studies in which positive animals were the young age animals (18, 30). In cats, we found that 66% (2/3) of the adult cats (between 1–3 years old) had positive samples of RVA. Our results align with a study in the UK that found no association between age and the prevalence of RVA in cats (33). Moreover, the occurrence of the viruses may depend on the time of sample collection, the age of the animals, and their clinical status.

Genotyping of RVA in this study showed that canine RVAs (*n* = 4) were identified as genotype G3P[3], which is consistent with the previously reported canine RVA genotype in Thailand (18). Our findings supported the evidence that G3P[3] is the predominant genotype circulating in domestic dogs in Thailand, similar to the worldwide prevalence of RVA genotype in dogs (7, 11, 34–36). It is noted that the G3P[3] genotype is still the only genotype ever reported in dogs. For the genotyping of feline RVA in cats, the Thai feline RVA (*n* = 1) was identified as genotype G3P[3]. Notably, the feline RVA genotype G3P[3] has been reported in cats worldwide except in Thailand. Apart from feline RVA genotype G3P[3], other genotypes ever reported in cats were genotype G3P[9] and G6P[9] (22, 33, 37). The RVA genotype G3P[3] has also been found in other animal species, such as in horses (E3198) and bats (LUS12-14, MSLH14) (27, 38, 39). It is interesting to note that the G3P[3] genotype has also been reported in humans in several countries, including the US (HCR3A), Israel (Ro1845), Italy (PA260-97), Brazil (R2638), Japan (12638), and Thailand (CMH222) (14, 17, 23, 25, 34).

### Genetic constellation of canine and feline RVAs

RVA genetic constellation based on the combination of 11 genotypes from each gene segment were analyzed (VP7-VP4-VP6-VP1-VP2-VP3-NSP1-NSP2-NSP3-NSP4-NSP5). Our result showed that two canine RVAs from dogs (CU25012, CU25170) were classified into genotype G3-P[3]-I3-R3-C3-M3-A9-N2-T3-E3-H6, which belongs to AU-1 genetic constellation. It is interesting to note that the canine RVAs genetic constellation had gene composition from both AU-1-like (VP2) and Cat-like genogroup (10 segments), the same as a previous study in Thailand (18). The VP2 genotype (C3) in canine RVAs in Thailand had never been reported in dogs in other countries, but it had been reported in RVAs from other species, such as cats, horses, bats, and humans, which could be the result of reassortment of the RVAs among animal species (22, 23, 27, 38). To the best of our knowledge, this specific genetic constellation has never been reported in dogs in other countries. However, a study conducted in Japan reported this genetic constellation in human RVA (12638), which could support the evidence of the potential for zoonotic transmission of this RVA in dogs (23).

For the Thai-feline RVA (CU25045), it was classified as genotype G3-P[3]-I8-R3-C3-M3-A9-N3-T3-E3-H6, which belongs to the AU-1 genetic constellation. This genetic constellation is unique due to its composition of gene segments derived from AU-1-like (VP2), Cat-like genogroup (9 segments), and an additional unique genotype of the VP6 gene segment (I8). This genetic pattern is unique and has never been reported in other RVAs in cats. From the nucleotide database, the VP6 (genotype I8) has never been reported in any RVAs from cats but has been found in bat RVAs from China (MSLH14, MYAS33) (27, 28). Interestingly, genotype I8 of the VP6 gene was found in human RVAs in Thailand, including CMH222, CMH079, and MS2015-1-0001 (24–26). Our result suggested that RVAs had been circulating among cats, bats, and humans. It is possible that reassortment events of feline RVAs have occurred within these animals and/or humans. This genetic constellation was also reported once in China’s bat strain MSLH14. This could support evidence of interspecies transmission between RVAs in cat and bat species (27).

### Reassortment event and zoonotic potential of canine and feline RVAs

**Figure 3:**
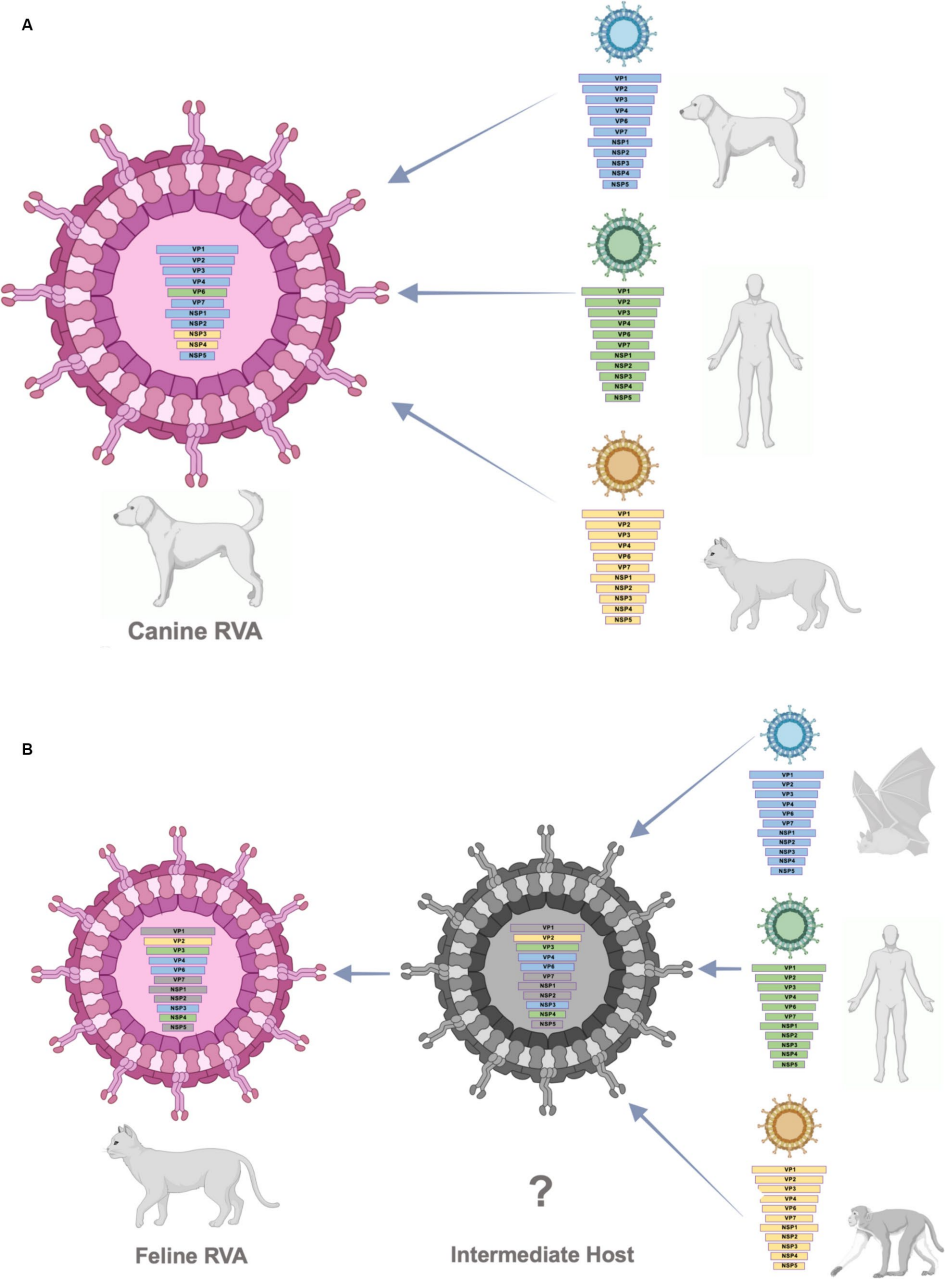
**(A)** Schematic presentation of possible multiple reassortments of Caine-RVAs in this study. **(B)** Schematic presentation of possible multiple reassortments of Feline RVAs in this study.

According to the result from phylogenetic analysis, nucleotide identities, and bootscan analysis, the canine RVAs in this study (CU25012, CU25170) may have resulted from multiple reassortments with dog, human, and cat RVAs (Figure 3). Our result indicated that 9 segments of canine RVAs were closely related to previously reported canine RVAs in Thailand, with 98–99% nucleotide and amino acid identities. While the VP6 gene was closely related to a human RVA (12638) from Japan, with 99% nucleotide and amino acid identities (23). The bootscan analysis also supported our finding that the VP6 gene might have originated from the reassortment of human RVAs. Interestingly, this human RVA (12638) contains an identical genetic constellation with canine RVAs, supporting the evidence of the zoonotic potential of canine RVAs. Moreover, the NSP3 gene of canine RVAs was closely related to feline RVA (FRV348), with 95–98% nucleotide and amino acid identities (22). Bootscan analysis also supported the reassortment event of the NSP3 and NSP4 genes from this feline RVA. Similar results of multiple reassortments of RVAs in dogs have also been reported in other studies, including the previous study in Thailand (18, 35). Moreover, there were many reports of canine-like RVAs genotype G3P[3] found in humans (8, 14, 17, 23). It should be noted that the inconclusive result of bootscan analysis in VP7, VP1, NSP1, and NSP2 genes might be due to reassortment from other unknown RVAs or intragenic recombination, which is rare but has been reported in some human studies (40–42). Thus, our result supported the evidence of reassortment events in canine RVAs and the zoonotic potential of the virus.

The result from phylogenetic analysis, nucleotide identities, and bootscan analysis showed that feline RVA (CU25045) was closely related to human, bat, and simian RVAs (Supplementary Figure S3B). The feline RVA was closely related to human RVAs in Thailand, including CMH079 (VP7 gene), CMH222 (VP4), MS2015-1-0001 (VP3, NSP2, NSP4, NSP5), with around 87–99% nucleotide and amino acid identities (25, 26). Bootscan analysis revealed that the reassortment event from human RVA (MS2015-1-0001) might occur in VP3 and NSP4 genes, indicating the potential for zoonotic transmission of RVAs. Unfortunately, human RVAs strains CMH222 and CMH079 were not included in the bootscan analysis due to incomplete whole genome sequences. For other gene segments, feline RVA was closely related to bat RVAs, MSLH14 (VP4), and MYAS33 (VP6, NSP1, NSP3, and NSP5), with around 87–100% nucleotide and amino acid identities (27, 28). While VP1 and VP2 genes of feline RVA were closely related to simian RVA (TUCH) reported in the USA, with 93–98% nucleotide and amino acid identities (29). The bootscan analysis supported the reassortment of bat RVAs, MSLH14 (VP4), and MYAS33 (VP6 and NSP3), as well as simian RVA, TUCH (VP2). It should be noted that even though the VP4 gene of feline RVA was closely related to human RVA (CMH222) and bat RVA (MSLH14), the nucleotide and amino acid identities were low compared to other segments, with only 87% (nt) and 95–97% (aa). This observation suggested that there might be a reassortment of VP4 from other unknown RVAs which did not include in the study. Interestingly, in this study, feline RVA was closely related to bat-like RVAs and had an identical genetic constellation as bat RVA (MSLH14) (27). The human RVA (MS2015-1-0001) found in Thailand was also closely related to feline RVA in this study and bat-like RVAs. This observation suggested the possible direct interspecies transmission of RVAs (26). On the other hand, human RVAs (CMH222, CMH079) and simian RVA (TUCH) showed the possibility of multiple reassortments from other animal species (24, 25, 29). Multiple reassortments of RVAs in cats had also been reported in previous studies (35, 37). Thus, the origin of feline RVA in this study is still inconclusive. It is speculated that direct interspecies transmission of the virus from other intermediate species to cats might occur, and the reassortment event might happen in those intermediate species (Supplementary Figure 3B). Since the study of RVAs in bats and simian species in Thailand is still limited, thus further studies of RVAs in bats, simians, and other wildlife species in Thailand are necessary to better understand the origin of RVAs and the genetic relationship among these species.

In conclusion, this study reported the genetic characteristics and diversity of RVAs in domestic dogs and cats in Thailand, which belong to the genotype G3P[3]. The canine RVAs showed evidence of multiple reassortments from canine, human, and feline RVAs. While feline RVA was closely related to RVAs in humans, bats, and simians and might have undergone direct interspecies transmission to cats. Our findings highlight the speculations regarding the zoonotic potential of both canine and feline RVAs. Furthermore, the study of RVAs in bats, simian, and other wildlife species in Thailand should be performed to understand the origin, genetic relationship, and genetic evolution among RVAs in animals.

## Data availability statement

The authors declare that the data supporting the findings of this study are available upon request from the first author. The nucleotide sequence data have been deposited at GenBank with accession numbers: OR501082-OR501118.

## Ethics statement

The animal studies were approved by Chulalongkorn University Animal Care and Use Committee. The studies were conducted in accordance with the local legislation and institutional requirements. Written informed consent was not obtained from the owners for the participation of their animals in this study because verbal consent was applied and used for sample collection per IACUC approval to compensate written informed consent.

## Author contributions

EC: Conceptualization, Investigation, Methodology, Visualization, Writing – original draft, Writing – review & editing. KC: Investigation, Methodology, Writing – original draft. KU: Investigation, Methodology, Writing – original draft. WJ: Investigation, Methodology, Writing – original draft. SC: Investigation, Methodology, Writing – original draft. AA: Conceptualization, Data curation, Formal analysis, Funding acquisition, Investigation, Methodology, Project administration, Resources, Supervision, Validation, Visualization, Writing – original draft, Writing – review & editing.

## Footnotes

^1^ https://www.genomedetective.com/app/typingtool/virus

^2^ https://www.viprbrc.org/brc/rvaGenotyper.spg
