# Supplementary material for: Genetic characterization and evidence for multiple reassortments of rotavirus A G3P[3] in dogs and cats in Thailand
Source: Front Vet Sci. 2024 May 24;11:1415771. doi: 10.3389/fvets.2024.1415771 (PMC11157116; doi:10.3389/fvets.2024.1415771)

**Supplement Materials**

**Genetic Characterization of Rotavirus A G3P[3] in Dogs and Cats:**

**Evidence for multiple reassortments and potential interspecies transmission**

Ekkapat Chamsai^1,2^, Kamonpan Charoenkul^1,2^, Kitikhun Udom^1,2^,

Waleemas Jairak^1,2^, Supassama Chaiyawong^1,2^, Alongkorn Amonsin^1,2^*

**Author affiliations**

^1^ Center of Excellence for Emerging and Re-emerging Infectious Diseases in Animals, and One Health Research Cluster, Faculty of Veterinary Science, Chulalongkorn University, Bangkok, Thailand
^2^ Department of Veterinary Public Health, Faculty of Veterinary Science, Chulalongkorn
University, Bangkok, Thailand

***Corresponding author:**

Professor Dr. Alongkorn Amonsin
**Mailing address:**

Center of Excellence for Emerging and Re-emerging Infectious Diseases in Animals,

Department of Veterinary Public Health, Faculty of Veterinary Science,
Chulalongkorn University, Bangkok, Thailand 10330
Phone: +66 2218 9578 Fax: +66 2218 9577
E-mail: alongkorn.a@chula.ac.th

**Supplement materials**

**Supplement Table**

Supplement Table 1: List of primers used for whole genome sequencing in this study.

Supplement Table 2. Nucleotide (nt) and amino acid (aa) identities between Canine RVA (CU25012) and reference RVAs.

Supplement Table 3. Nucleotide (nt) and amino acid (aa) identities between Feline RVA (CU25045) and reference RVAs.

**Supplement Figure**

Supplement Figure 1. Phylogenetic trees of VP1, VP2, VP3, VP6 genes of canine RVAs and feline RVAs characterized in this study.

Supplement Figure 2. Phylogenetic trees of NS1, NS2, NS3, NS4, NP5 genes of canine RVAs and feline RVAs characterized in this study.

Supplement Table 1: List of primers used for whole genome sequencing in this study.

| Primer name | Type | Sequence | Gene | Position(nt) | Product size(bp) | Annealing temp. | Reference |
| --- | --- | --- | --- | --- | --- | --- | --- |
| RVA_VP1F_out | F | GGCTATTWAAGCTGTACAATGG | VP1 | 1-3300 | 3300 | 48°C | Charoenkul et al. 2020 |
| RVA_VP1R_out | R | GGTCACATCYAAGCACTC |  |  |  |  |  |
| RVA_VP2F_out | F | GGCTATTAAAGGYTCAATG | VP2 | 1-2700 | 2700 | 48°C | Charoenkul et al. 2020 |
| RVA_VP2R_out | R | GGTCATATCTCCACAGTG |  |  |  |  |  |
| RVA_VP3F_out | F | GGCTWTTAAAGCAGTATGAG | VP3 | 1-2581 | 2581 | 48°C | Charoenkul et al. 2020 |
| RVA_VP3R_out | R | GGTCAMATCGTGACTAG |  |  |  |  |  |
| RVA_VP4F_out | F | GGCTATAAAATGGCTTCGCTCATT | VP4 | 1-2300 | 2300 | 48°C | Charoenkul et al. 2020 |
| RVA_VP4R_out | R | GGTCACATCCTCTAGAAATTGC |  |  |  |  |  |
| RVA_VP6F_out | F | GGCTTTTAAACGAAGTCTTC | VP6 | 1-1350 | 1350 | 48°C | Charoenkul et al. 2020 |
| RVA_VP6R_out | R | GGTCACATCCTCTCACT |  |  |  |  |  |
| RVA_VP7F_out | F | GGCTTTAAAAGCGAGAATTTCCGT | VP7 | 1-1036 | 1036 | 48°C | Charoenkul et al. 2020 |
| RVA_VP7R_out | R | GGTCACATCATACAATTCTAA |  |  |  |  |  |
| RVA_NSP1F_out | F | GGCTTTTTTTTGAAAAGTCTTG | NSP1 | 1-1500 | 1500 | 50°C | Charoenkul et al. 2020 |
| RVA_NSP1R_out | R | GGTTCACAGTTTTTGCTGGCTAGG |  |  |  |  |  |
| RVA_NSP2F_out | F | GGCTTTTAAAGCGTCTCAG | NSP2 | 1-1021 | 1021 | 48°C | Charoenkul et al. 2020 |
| RVA_NSP2R_out | R | GGTCACATAAGCGCTTTC |  |  |  |  |  |
| RVA_NSP3F_out | F | GGCTTTTAATGCTTTTCAGTG | NSP3 | 1-1100 | 1100 | 50°C | Charoenkul et al. 2020 |
| RVA_NSP3R_out | R | GGTCACATAACGCCCCTATAGC |  |  |  |  |  |
| RVA_NSP4F_out | F | GGCTTTTAAAAGTTCTGTTCCG | NSP4 | 1-750 | 750 | 53°C | Charoenkul et al. 2020 |
| RVA_NSP4R_out | R | GGTCACAYAAAGACCGTTCCTTCC |  |  |  |  |  |
| RVA_NSP5F_out | F | GGCTTTTAAAGCGCTACAG | NSP5 | 1-780 | 780 | 48°C | Charoenkul et al. 2020 |
| RVA_NSP5R_out | R | GGTCACAAAACGGGAGT |  |  |  |  |  |
| RVA_VP4_con3 | F | TGGCTTCGCTCATTTATAGACA | VP4 | 31-871 | 840 | 45°C | Gentsch et al. 2014 |
| RVA_VP4_con2 | R | ATTTCGGACCATTTATAACC |  |  |  |  |  |

Supplement Table 2. Nucleotide (nt) and amino acid (aa) identities between Canine RVA (CU25012) and reference RVAs.

| **Virus** | **Strain** | **Species** | **Year** | **Country** | | **Nucleotide (nt) and Amino acid (aa) identities (%)** | | | | | | | | | | | |
| --- | --- | --- | --- | --- | --- | --- | --- | --- | --- | --- | --- | --- | --- | --- | --- | --- | --- |
|  |  |  |  |  |  | **Gene** | | | | | | | | | | | |
|  |  |  |  |  |  | **VP7** | | **VP4** | | **VP6** | | **VP1** | | **VP2** | | **VP3** | |
|  |  |  |  |  |  | **nt** | **aa** | **nt** | **aa** | **nt** | **aa** | **nt** | **aa** | **nt** | **aa** | **nt** | **aa** |
| **RVA/Dog/THA/CU25012/2020/G3P[3]** | **RV25012** | **Dog** | **2020** | **THA** |  | |  |  |  |  |  |  |  |  |  |  |  |
| **RVA/Dog/THA/CU25170/2020/G3P[3]** | **DC25170** | **Dog** | **2020** | **THA** | 99.90 | | 100.00 | 99.91 | 100.00 | 99.83 | 99.74 | NA | NA | 99.88 | 100.00 | 99.96 | 100.00 |
| RVA/Dog-tc/USA/A79-10/1979/G3P[3] | A79-10 | Dog | 1979 | USA | 95.59 | | 98.75 | 93.77 | 97.35 | 84.76 | 96.50 | 86.12 | 95.40 | 82.08 | 96.72 | 84.93 | 91.28 |
| RVA/Dog-tc/USA/K9/1981/G3P[3] | K9 | Dog | 1981 | USA | 94.46 | | 98.44 | 95.10 | 96.67 | 84.33 | 96.50 | 85.58 | 95.00 | 82.04 | 96.72 | 85.01 | 91.70 |
| RVA/Dog-tc/USA/CU-1/1982/G3P[3] | CU-1 | Dog | 1982 | USA | 95.59 | | 98.75 | 93.43 | 96.40 | 84.59 | 96.77 | 86.12 | 95.20 | 81.80 | 96.46 | 85.29 | 91.42 |
| RVA/Dog-tc/JPN/RS15/1982/G3P[3] | RS15 | Dog | 1982 | JPN | 93.74 | | 98.75 | 95.40 | 97.22 | 85.19 | 97.87 | 86.12 | 95.40 | 82.23 | 96.72 | 85.09 | 91.56 |
| RVA/Dog-tc/ITA/RV198-95/1995/G3P[3] | RV198-95 | Dog | 1995 | ITA | 94.05 | | 98.75 | 94.12 | 96.67 | 96.97 | 99.48 | 86.00 | 95.20 | 81.88 | 96.84 | 97.05 | 97.92 |
| RVA/Dog-tc/ITA/RV52-96/1996/G3P[3] | RV52-96 | Dog | 1996 | ITA | 84.31 | | 93.79 | 96.65 | 97.08 | 96.10 | 98.95 | 86.00 | 95.20 | 82.27 | 96.21 | 97.17 | 98.29 |
| RVA/Dog/THA/CU132/2017/G3P[3] | CU132 | Dog | 2017 | THA | 98.26 | | 99.38 | 98.37 | 99.09 | 98.96 | 99.74 | 98.72 | 99.45 | 98.75 | 99.65 | 98.68 | 99.03 |
| RVA/Dog/THA/CU23379/2019/G3P[3] | CU23379 | Dog | 2019 | THA | 97.33 | | 98.75 | 98.11 | 98.96 | 99.05 | 99.48 | 98.48 | 99.26 | 98.28 | 99.88 | 98.37 | 99.28 |
| RVA/Cat-tc/AUS/Cat97/1984/G3P[3] | Cat97 | Cat | 1984 | AUS | 94.05 | | 98.75 | 93.99 | 96.67 | 84.68 | 96.50 | 85.94 | 94.80 | 81.61 | 96.59 | 84.73 | 91.84 |
| RVA/Cat/JPN/FRV348/1994/G3P[3] | FRV348 | Cat | 1994 | JPN | 84.31 | | 94.48 | 97.16 | 97.76 | 96.97 | 99.48 | 93.61 | 97.56 | 96.21 | 99.65 | 97.21 | 98.54 |
| RVA/Bat-wt/ZMB/LUS12-14/2012/G3P[3] | LUS12-14 | Bat | 2012 | ZMB | 84.00 | | 93.79 | 97.51 | 98.43 | 94.46 | 99.21 | 80.10 | 93.57 | 81.69 | 96.97 | 98.52 | 99.03 |
| RVA/Bat-wt/CHN/MSLH14/2012/G3P[3] | MSLH14 | Bat | 2012 | CHN | 85.64 | | 95.16 | 81.62 | 91.47 | 82.08 | 96.50 | 84.54 | 95.80 | 87.54 | 99.53 | 87.24 | 94.17 |
| RVA/Bat-wt/CHN/MYAS33/2012/G3P[10] | MYAS33 | Bat | 2013 | CHN | 85.64 | | 95.83 | 75.77 | 84.52 | 82.51 | 96.77 | 87.40 | 96.20 | 88.52 | 99.41 | 86.80 | 93.77 |
| RVA/Bat-wt/CHN/LZHP2/2015/G3P[3] | LZHP2 | Bat | 2015 | CHN | 84.92 | | 94.82 | 81.01 | 87.21 | 85.37 | 97.33 | 85.15 | 93.15 | 87.04 | 95.96 | 87.32 | 90.99 |
| RVA/Human-tc/USA/HCR3A/1984/G3P[3] | HCR3A | Human | 1984 | USA | 94.97 | | 99.07 | 94.80 | 96.53 | 84.50 | 96.77 | 85.64 | 95.40 | 81.96 | 96.84 | 85.17 | 91.84 |
| RVA/Human-tc/ISR/Ro1845/1985/G3P[3] | Ro1845 | Human | 1985 | ISR | 94.26 | | 98.75 | 93.73 | 96.67 | 84.94 | 96.77 | 85.51 | 95.40 | 81.84 | 96.97 | 84.61 | 90.99 |
| RVA/Human-tc/ITA/PA260-97/1997/G3P[3] | PA260-97 | Human | 1997 | ITA | 83.90 | | 93.79 | 96.82 | 97.49 | 96.97 | 99.48 | 93.73 | 97.94 | 95.08 | 99.53 | 97.05 | 97.92 |
| RVA/Human-wt/JPN/12638/2014/G3P[3] | 12638 | Human | 2014 | JPN | 98.56 | | 99.38 | 92.91 | 96.26 | 99.05 | 99.48 | 98.05 | 99.63 | 97.07 | 99.76 | 94.06 | 97.42 |

| **Virus** | **Strain** | **Species** | **Year** | | **Country** | **Nucleotide (nt) and Amino acid (aa) identities (%)** | | | | | | | | | |
| --- | --- | --- | --- | --- | --- | --- | --- | --- | --- | --- | --- | --- | --- | --- | --- |
|  |  |  |  |  |  | **Gene** | | | | | | | | | |
|  |  |  |  |  |  | **NSP1** | | **NSP2** | | **NSP3** | | **NSP4** | | **NSP5** | |
|  |  |  |  |  |  | **nt** | **aa** | **nt** | **aa** | **nt** | **aa** | **nt** | **aa** | **nt** | **aa** |
| **RVA/Dog/THA/CU25012/2020/G3P[3]** | **RV25012** | **Dog** | **2020** | **THA** | |  |  |  |  |  |  |  |  |  |  |
| **RVA/Dog/THA/CU25170/2020/G3P[3]** | **DC25170** | **Dog** | **2020** | **THA** | | 99.73 | 99.79 | 99.90 | 100.00 | 99.89 | 100.00 | 99.62 | 100.00 | 100.00 | 100.00 |
| RVA/Dog-tc/USA/A79-10/1979/G3P[3] | A79-10 | Dog | 1979 | USA | | 83.41 | 85.41 | 84.91 | 92.54 | 85.77 | 92.07 | 84.28 | 92.64 | 93.97 | 95.24 |
| RVA/Dog-tc/USA/K9/1981/G3P[3] | K9 | Dog | 1981 | USA | | 82.73 | 84.60 | 85.74 | 92.54 | 85.56 | 92.07 | 84.28 | 92.64 | 93.47 | 94.12 |
| RVA/Dog-tc/USA/CU-1/1982/G3P[3] | CU-1 | Dog | 1982 | USA | | 82.87 | 85.14 | 85.53 | 92.91 | 85.46 | 91.32 | 84.28 | 93.29 | 94.64 | 95.24 |
| RVA/Dog-tc/JPN/RS15/1982/G3P[3] | RS15 | Dog | 1982 | JPN | | 82.73 | 84.60 | 80.71 | 86.79 | 86.52 | 92.81 | 91.86 | 95.21 | 94.81 | 95.24 |
| RVA/Dog-tc/ITA/RV198-95/1995/G3P[3] | RV198-95 | Dog | 1995 | ITA | | 83.14 | 85.14 | 96.65 | 98.40 | 86.84 | 92.07 | 84.09 | 92.64 | 94.30 | 94.68 |
| RVA/Dog-tc/ITA/RV52-96/1996/G3P[3] | RV52-96 | Dog | 1996 | ITA | | 82.66 | 84.32 | 97.27 | 98.72 | 86.41 | 92.07 | 98.30 | 98.84 | 93.63 | 94.68 |
| RVA/Dog/THA/CU132/2017/G3P[3] | CU132 | Dog | 2017 | THA | | 98.57 | 99.38 | 98.32 | 99.37 | 90.98 | 95.32 | 99.05 | 98.84 | 99.50 | 98.98 |
| RVA/Dog/THA/CU23379/2019/G3P[3] | CU23379 | Dog | 2019 | THA | | 98.09 | 98.96 | 97.80 | 99.68 | 90.98 | 94.97 | 98.67 | 98.84 | 99.33 | 98.98 |
| RVA/Cat-tc/AUS/Cat97/1984/G3P[3] | Cat97 | Cat | 1984 | AUS | | 82.94 | 84.32 | 85.32 | 92.54 | 86.41 | 92.44 | 84.28 | 92.64 | 93.63 | 93.55 |
| RVA/Cat/JPN/FRV348/1994/G3P[3] | FRV348 | Cat | 1994 | JPN | | 43.28 | 52.90 | 80.82 | 86.79 | 95.65 | 98.05 | 97.73 | 97.66 | 94.14 | 95.79 |
| RVA/Bat-wt/ZMB/LUS12-14/2012/G3P[3] | LUS12-14 | Bat | 2012 | ZMB | | 98.16 | 98.75 | 97.90 | 98.40 | 90.13 | 94.97 | 78.03 | 80.14 | 89.28 | 91.80 |
| RVA/Bat-wt/CHN/MSLH14/2012/G3P[3] | MSLH14 | Bat | 2012 | CHN | | 86.69 | 92.26 | 80.29 | 87.19 | 87.37 | 92.81 | 88.45 | 93.94 | 93.30 | 96.88 |
| RVA/Bat-wt/CHN/MYAS33/2012/G3P[10] | MYAS33 | Bat | 2013 | CHN | | 87.44 | 92.73 | 80.61 | 87.59 | 86.62 | 94.61 | 87.12 | 93.94 | 93.97 | 97.41 |
| RVA/Bat-wt/CHN/LZHP2/2015/G3P[3] | LZHP2 | Bat | 2015 | CHN | | 87.03 | 90.56 | 81.24 | 86.79 | 85.56 | 92.81 | 91.29 | 94.58 | 92.96 | 95.79 |
| RVA/Human-tc/USA/HCR3A/1984/G3P[3] | HCR3A | Human | 1984 | USA | | 82.73 | 84.60 | 85.74 | 92.91 | 85.56 | 92.44 | 83.71 | 92.64 | 94.30 | 94.68 |
| RVA/Human-tc/ISR/Ro1845/1985/G3P[3] | Ro1845 | Human | 1985 | ISR | | 82.87 | 84.60 | 85.64 | 92.91 | 86.09 | 93.17 | 84.47 | 92.64 | 93.80 | 93.55 |
| RVA/Human-tc/ITA/PA260-97/1997/G3P[3] | PA260-97 | Human | 1997 | ITA | | 43.07 | 50.36 | 97.17 | 99.04 | 85.67 | 91.70 | 97.73 | 98.84 | 94.30 | 95.24 |
| RVA/Human-wt/JPN/12638/2014/G3P[3] | 12638 | Human | 2014 | JPN | | 98.23 | 98.54 | 97.48 | 98.40 | 90.76 | 95.32 | 97.16 | 98.84 | 98.16 | 98.46 |

Supplement Table 3. Nucleotide (nt) and amino acid (aa) identities between Feline RVA (CU25045) and reference RVAs.

| **Virus** | **Strain** | **Species** | **Year** | **Country** | **Nucleotide (nt) and Amino acid (aa) identities (%)** | | | | | | | | | | | |
| --- | --- | --- | --- | --- | --- | --- | --- | --- | --- | --- | --- | --- | --- | --- | --- | --- |
|  |  |  |  |  | **Gene** | | | | | | | | | | | |
|  |  |  |  |  | **VP7** | | **VP4** | | **VP6** | | **VP1** | | **VP2** | | **VP3** | |
|  |  |  |  |  | **nt** | **aa** | **nt** | **aa** | **nt** | **aa** | **nt** | **aa** | **nt** | **aa** | **nt** | **aa** |
| **RVA/Cat/THA/CU25045/2020/G3P[3]** | **RV25045** | **Cat** | **2020** | **THA** |  |  |  |  |  |  |  |  |  |  |  |  |
| RVA/Cat-tc/AUS/Cat97/1984/G3P[3] | Cat97 | Cat | 1984 | AUS | 85.44 | 95.83 | 81.05 | 91.21 | 82.51 | 95.93 | 86.02 | 96.22 | 81.25 | 95.72 | 82.81 | 91.70 |
| RVA/Cat-tc/AUS/Cat2/1984/G3P[9] | Cat2 | Cat | 1984 | AUS | 82.36 | 92.03 | 67.71 | 58.75 | 81.21 | 97.33 | 85.95 | 96.02 | 81.48 | 95.84 | 82.62 | 90.99 |
| RVA/Cat/JPN/FRV348/1994//G3P[3] | FRV348 | Cat | 1994 | JPN | 88.10 | 96.50 | 81.10 | 91.36 | 81.99 | 96.77 | 88.79 | 97.41 | 88.12 | 98.14 | 87.64 | 93.90 |
| RVA/Cat/JPN/FRV384/1994//G3P[3] | FRV384 | Cat | 1994 | JPN | 81.44 | 92.03 | 67.54 | 58.75 | 80.87 | 97.05 | 87.04 | 96.52 | 87.48 | 98.38 | 85.73 | 92.54 |
| RVA/Cat-wt/ITA/BA222/2005/G3P[9] | BA222 | Cat | 2005 | ITA | 81.44 | 91.30 | 67.62 | 59.27 | 81.21 | 95.37 | 79.73 | 93.64 | 80.65 | 95.10 | 76.20 | 81.73 |
| RVA/Bat-wt/CHN/MSLH14/2012/G3P[3] | MSLH14 | Bat | 2012 | CHN | 88.72 | 97.48 | 87.61 | 97.60 | 87.88 | 99.48 | 84.05 | 96.62 | 86.34 | 98.38 | 92.07 | 96.66 |
| RVA/Bat-wt/CHN/MYAS33/2012/G3P[3] | MYAS33 | Bat | 2013 | CHN | 89.13 | 98.12 | 77.71 | 87.70 | 95.84 | 100.00 | 93.02 | 98.10 | 88.65 | 98.38 | 92.03 | 96.40 |
| RVA/Human-wt/THA/CMH222/2001/G3P[3] | CMH222 | Human | 2001 | THA | 97.03 | 98.75 | 87.66 | 95.79 | 83.29 | 98.14 | NA | NA | NA | NA | NA | NA |
| RVA/Human-wt/THA/CMH079/2005/G3P[10] | CMH079 | Human | 2005 | THA | 97.23 | 99.07 | NA | NA | 94.89 | 98.95 | NA | NA | NA | NA | NA | NA |
| RVA/Human-tc/THA/CU-365/2008/G3P[9] | CU365 | Human | 2008 | THA | 82.15 | 92.03 | 67.54 | 59.52 | 81.73 | 97.60 | 88.10 | 96.52 | 87.21 | 98.38 | 87.24 | 94.04 |
| RVA/Human-wt/JPN/12638/2014/G3P[3] | 12638 | Human | 2014 | JPN | 84.62 | 94.48 | 81.44 | 90.74 | 81.13 | 96.77 | 88.10 | 97.21 | 88.61 | 98.38 | 87.60 | 94.17 |
| RVA/Human-wt/THA/MS2015-1-0001/G3P10 | MS2015-1-0001 | Human | 2015 | THA | 88.38 | 97.16 | 77.52 | 87.54 | 93.47 | 100.00 | 92.10 | 98.13 | 88.40 | 97.93 | 94.06 | 97.04 |
| RVA/Rhesus-tc/USA/TUCH/2002/G3P24 | TUCH | Simian | 2002 | USA | 79.10 | 87.97 | 76.75 | 85.71 | 81.32 | 95.53 | 93.17 | 97.84 | 92.83 | 98.63 | 85.77 | 92.95 |

| **Virus** | **Strain** | **Species** | **Year** | **Country** | **Nucleotide (nt) and Amino acid (aa) identities (%)** | | | | | | | | | | | | | | | |
| --- | --- | --- | --- | --- | --- | --- | --- | --- | --- | --- | --- | --- | --- | --- | --- | --- | --- | --- | --- | --- |
|  |  |  |  |  | **Gene** | | | | | | | | | | | | | | | |
|  |  |  |  |  | **NSP1** | | | **NSP2** | | **NSP3** | | | **NSP4** | | | | **NSP5** | | | |
|  |  |  |  |  | **nt** | | **aa** | **nt** | **aa** | **nt** | **aa** | | **nt** | | **aa** | | **nt** | | **aa** | |
| **RVA/Cat/THA/CU25045/2020/G3P[3]** | **RV25045** | **Cat** | **2020** | **THA** | |  |  |  |  |  | |  | |  | |  | |  | |  |
| RVA/Cat-tc/AUS/Cat97/1984/G3P[3] | Cat97 | Cat | 1984 | AUS | | 82.67 | 58.94 | 79.56 | 87.59 | 84.29 | | 93.90 | | 84.47 | | 93.94 | | 93.47 | | 95.24 |
| RVA/Cat-tc/AUS/Cat2/1984/G3P[9] | Cat2 | Cat | 1984 | AUS | | 50.59 | NA | 80.61 | 89.16 | 76.75 | | 79.62 | | 85.23 | | 95.21 | | 89.78 | | 93.55 |
| RVA/Cat/JPN/FRV348/1994//G3P[3] | FRV348 | Cat | 1994 | JPN | | 51.21 | NA | 88.68 | 97.41 | 87.58 | | 96.70 | | 88.83 | | 97.06 | | 92.63 | | 96.34 |
| RVA/Cat/JPN/FRV384/1994//G3P[3] | FRV384 | Cat | 1994 | JPN | | 50.87 | NA | 91.51 | 96.74 | 85.46 | | 95.67 | | 89.39 | | 98.84 | | 88.44 | | 91.21 |
| RVA/Cat-wt/ITA/BA222/2005/G3P[9] | BA222 | Cat | 2005 | ITA | | 50.31 | NA | 79.66 | 85.97 | 85.88 | | 95.67 | | 78.22 | | 84.11 | | 88.94 | | 91.80 |
| RVA/Bat-wt/CHN/MSLH14/2012/G3P[3] | MSLH14 | Bat | 2012 | CHN | | 92.38 | 88.59 | 91.19 | 98.40 | 86.31 | | 94.61 | | 94.32 | | 98.26 | | 96.15 | | 97.94 |
| RVA/Bat-wt/CHN/MYAS33/2012/G3P[3] | MYAS33 | Bat | 2013 | CHN | | 94.04 | 90.24 | 90.78 | 98.40 | 96.07 | | 97.38 | | 90.53 | | 98.26 | | 96.82 | | 98.46 |
| RVA/Human-wt/THA/CMH222/2001/G3P[3] | CMH222 | Human | 2001 | THA | | NA | NA | NA | NA | NA | | NA | | 87.69 | | 99.43 | | NA | | NA |
| RVA/Human-wt/THA/CMH079/2005/G3P[10] | CMH079 | Human | 2005 | THA | | NA | NA | NA | NA | NA | | NA | | 89.58 | | 97.06 | | 93.80 | | 97.41 |
| RVA/Human-tc/THA/CU-365/2008/G3P[9] | CU365 | Human | 2008 | THA | | 49.69 | NA | 90.99 | 95.72 | 89.07 | | 98.38 | | 88.64 | | 96.45 | | 93.63 | | 96.88 |
| RVA/Human-wt/JPN/12638/2014/G3P[3] | 12638 | Human | 2014 | JPN | | 86.63 | 74.66 | 81.55 | 87.59 | 86.20 | | 95.67 | | 89.02 | | 97.06 | | 93.80 | | 98.46 |
| RVA/Human-wt/THA/MS2015-1-0001/G3P[10] | MS2015-1-0001 | Human | 2015 | THA | | 88.64 | 92.58 | 92.66 | 99.04 | 87.26 | | 96.36 | | 98.11 | | 98.84 | | 96.82 | | 98.46 |
| RVA/Rhesus-tc/USA/TUCH/2002/G3P[24] | TUCH | Simian | 2002 | USA | | 86.68 | 92.11 | 81.45 | 88.38 | 92.25 | | 95.67 | | 84.28 | | 93.94 | | 94.30 | | 98.98 |

Supplement Figure 1. Phylogenetic trees of VP1, VP2, VP3, VP6 genes of canine RVAs and feline RVAs characterized in this study.

**VP1**


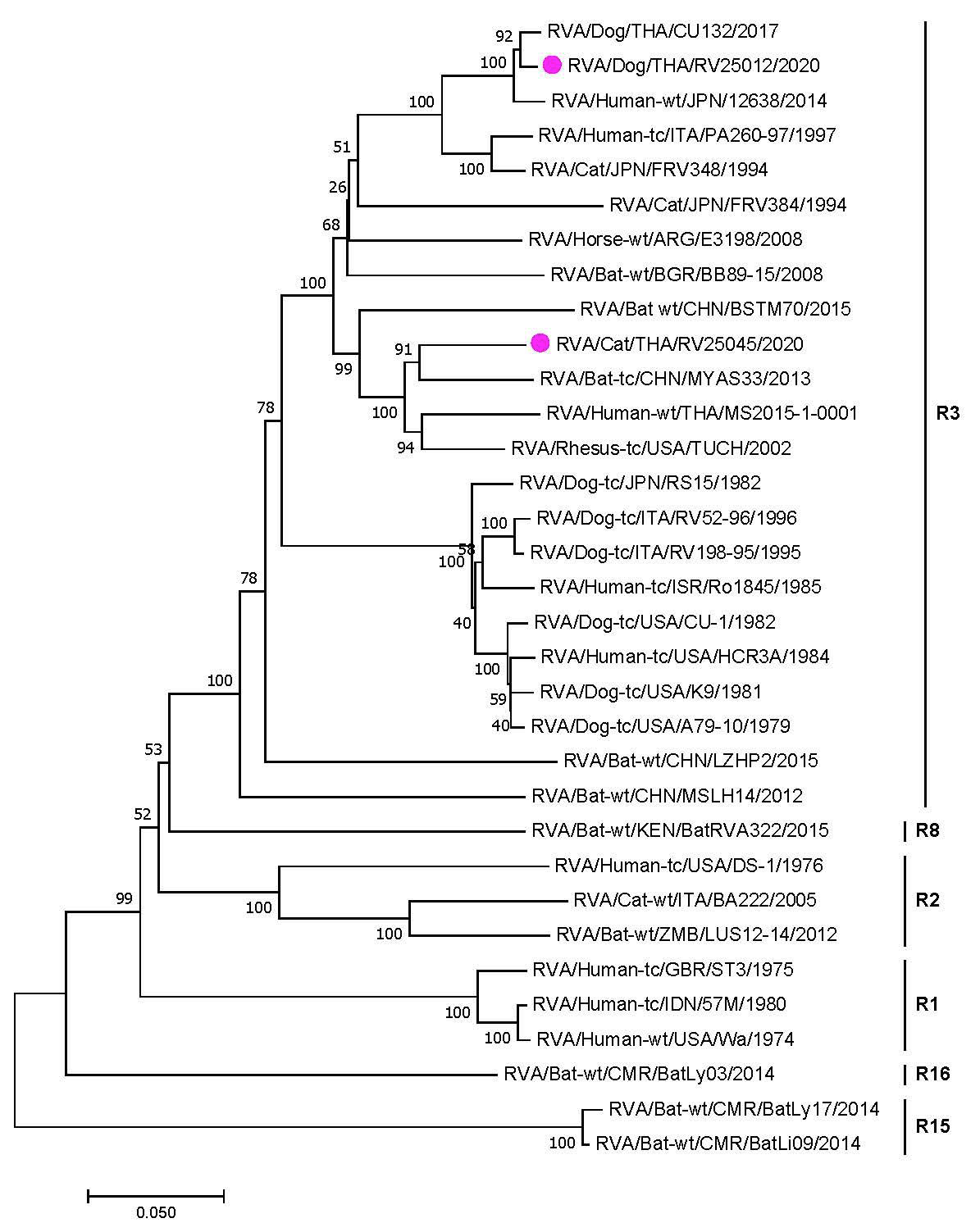


**VP2**

**
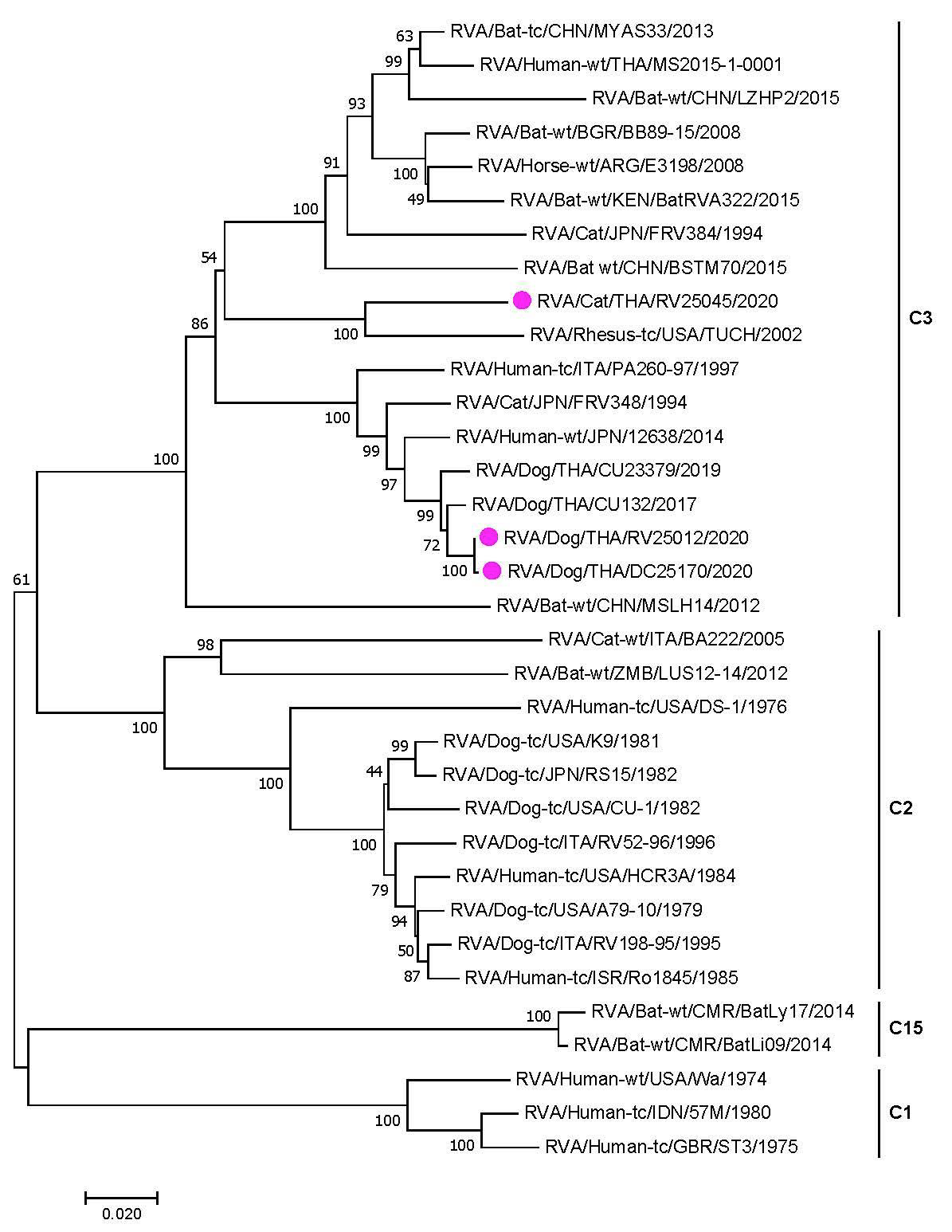
**


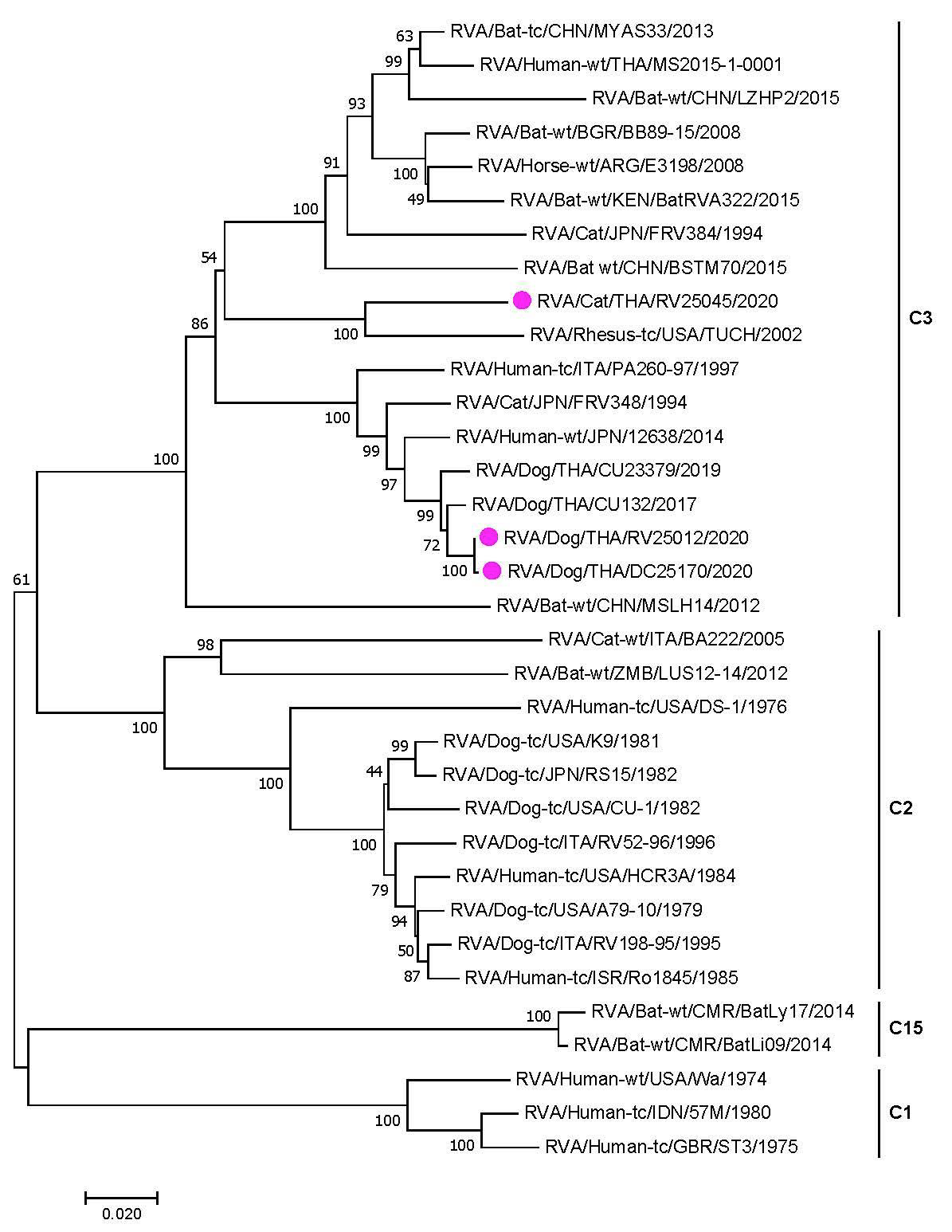


**VP3**


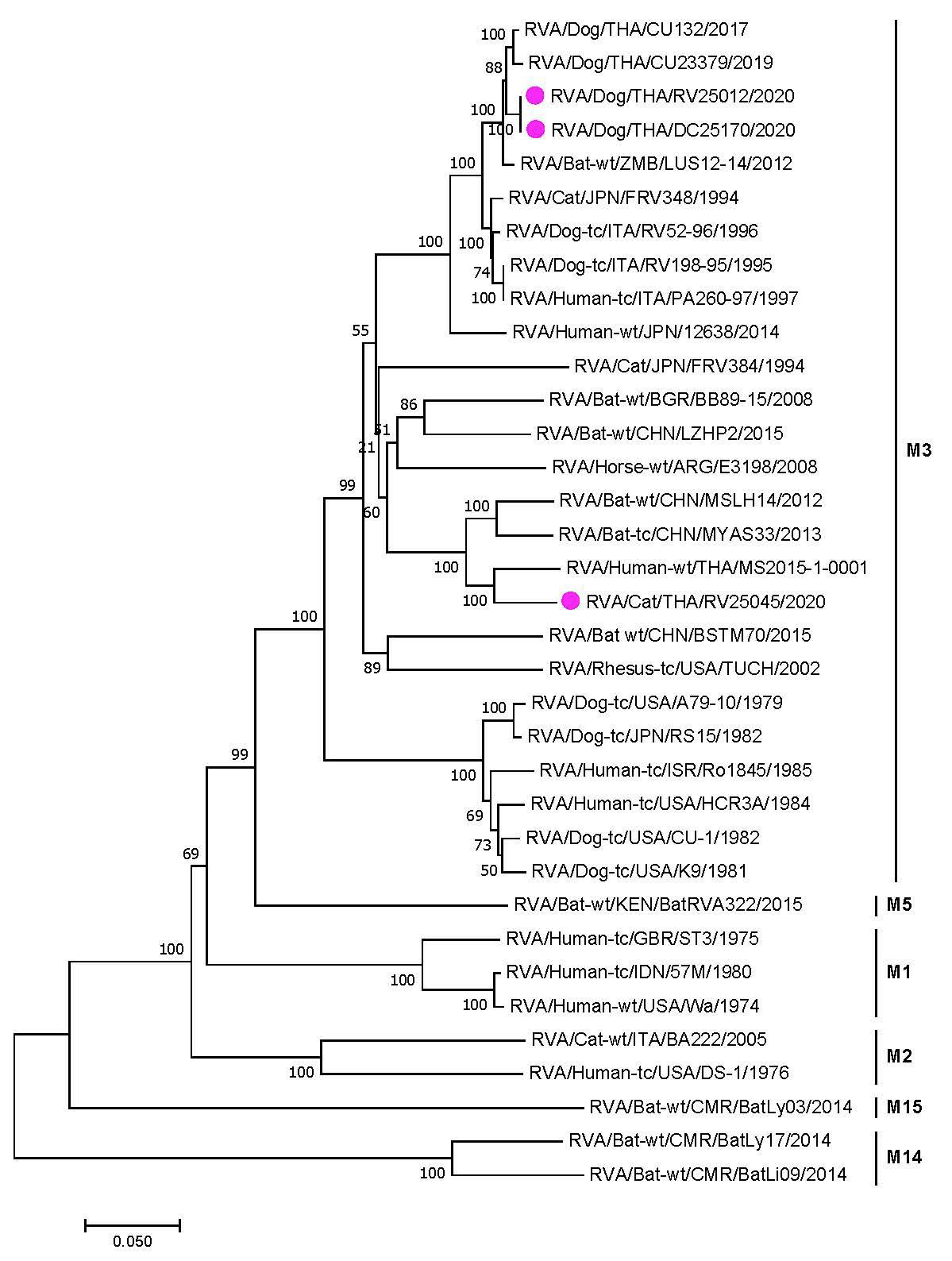


**VP6**


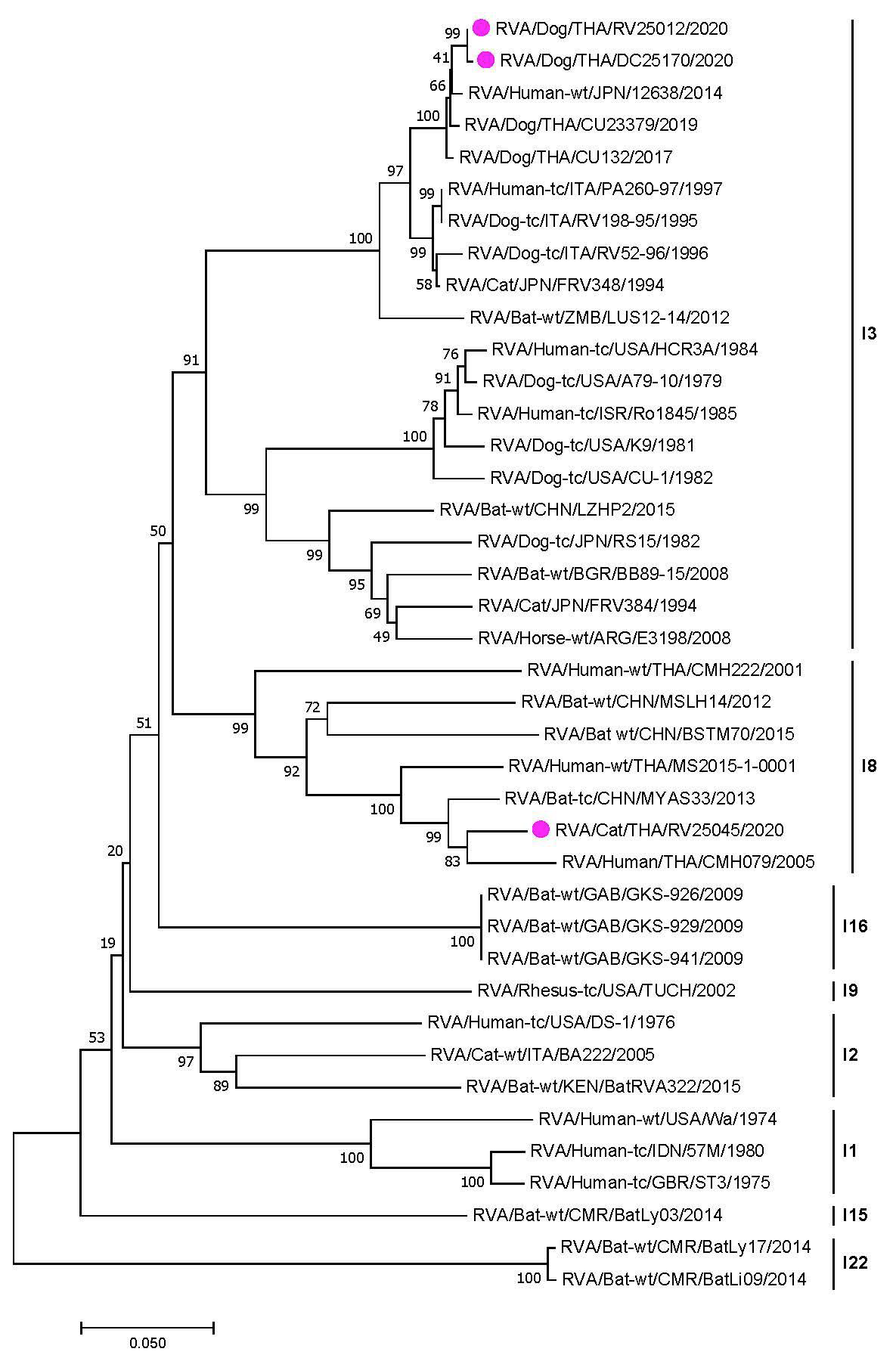


Supplement Figure 2. Phylogenetic trees of NS1, NS2, NS3, NS4, NP5 genes of canine RVAs and feline RVAs characterized in this study.

**NSP1**


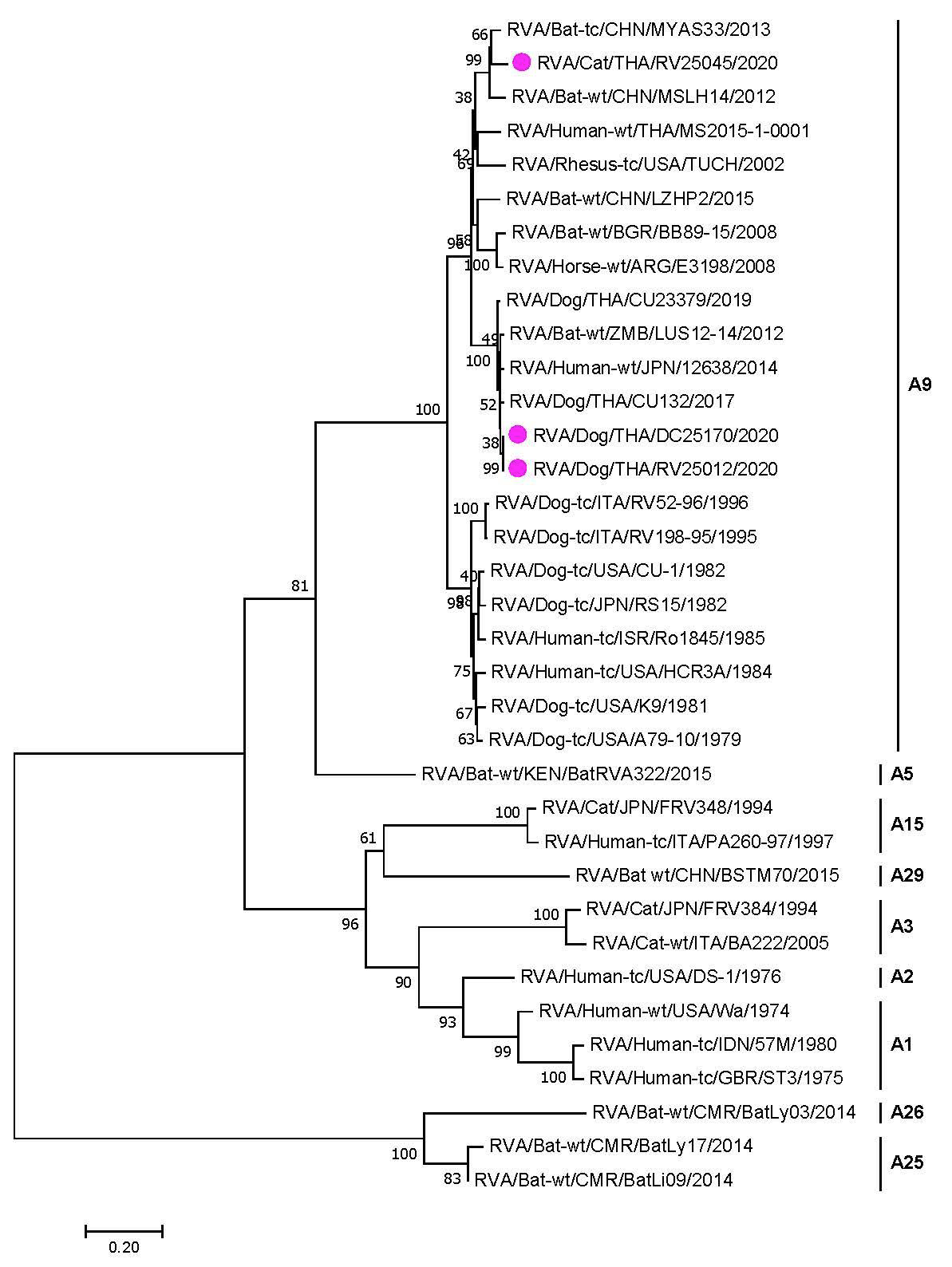


**NSP2**


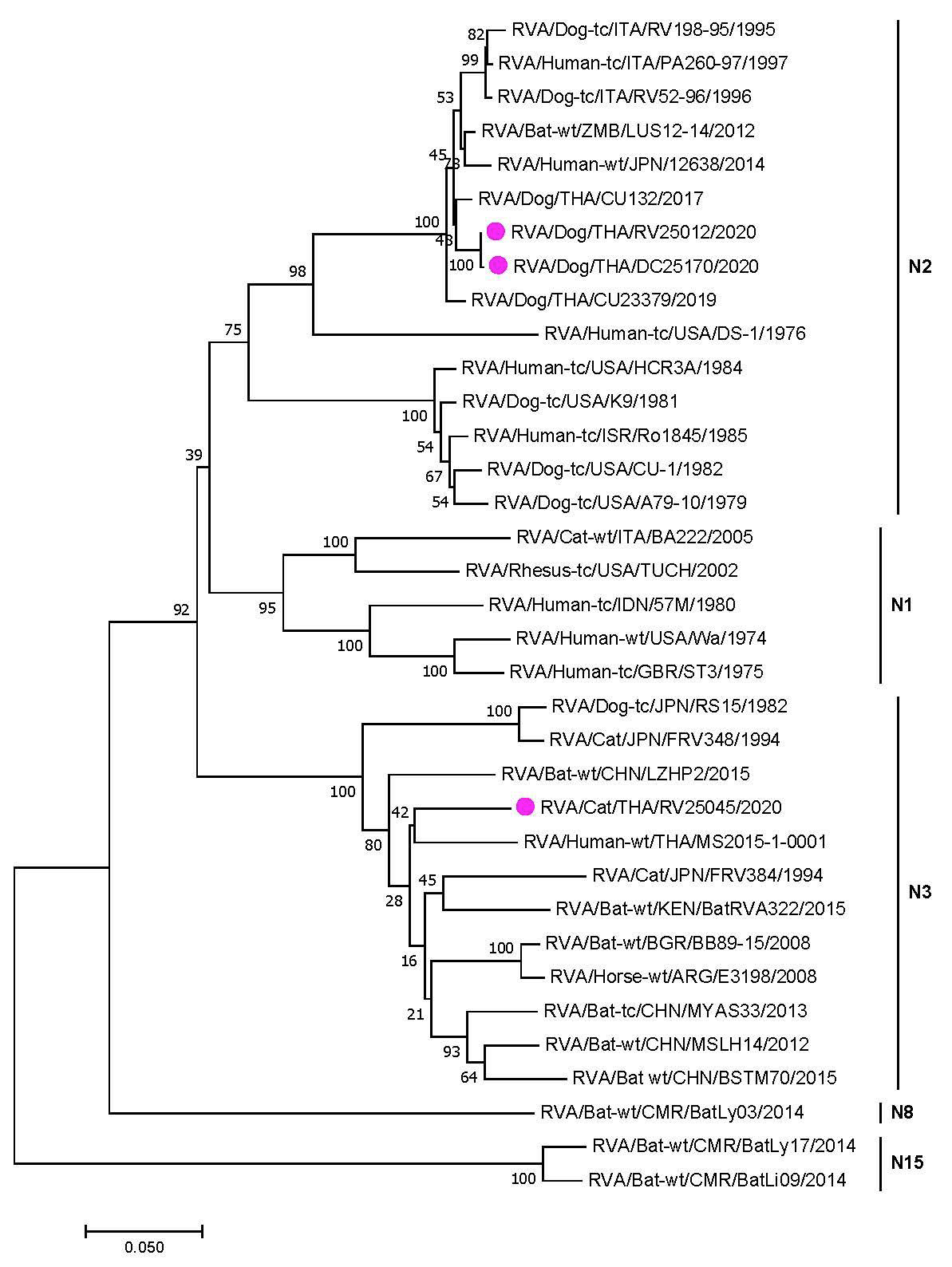


**NSP3**


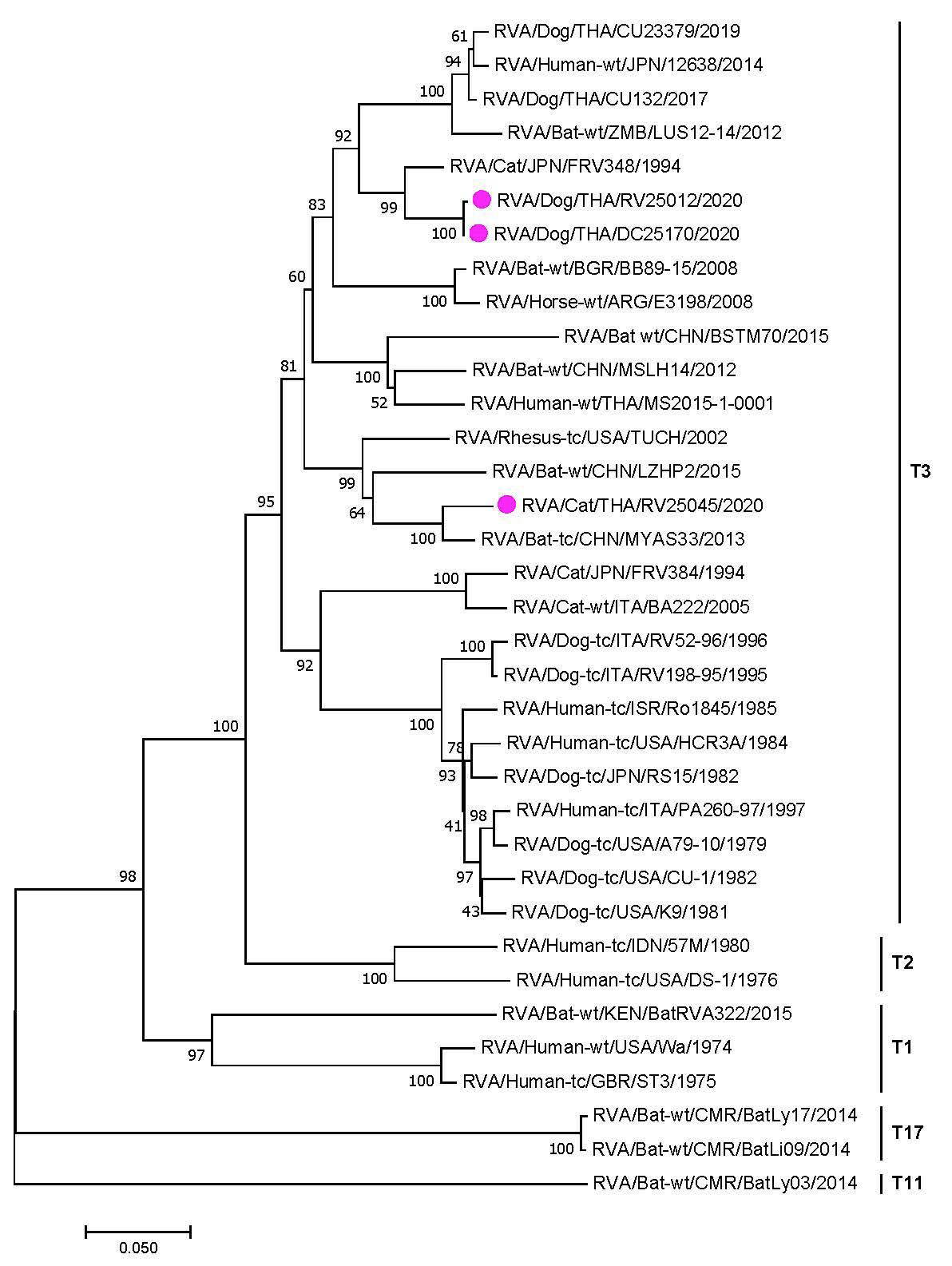


**NSP4**


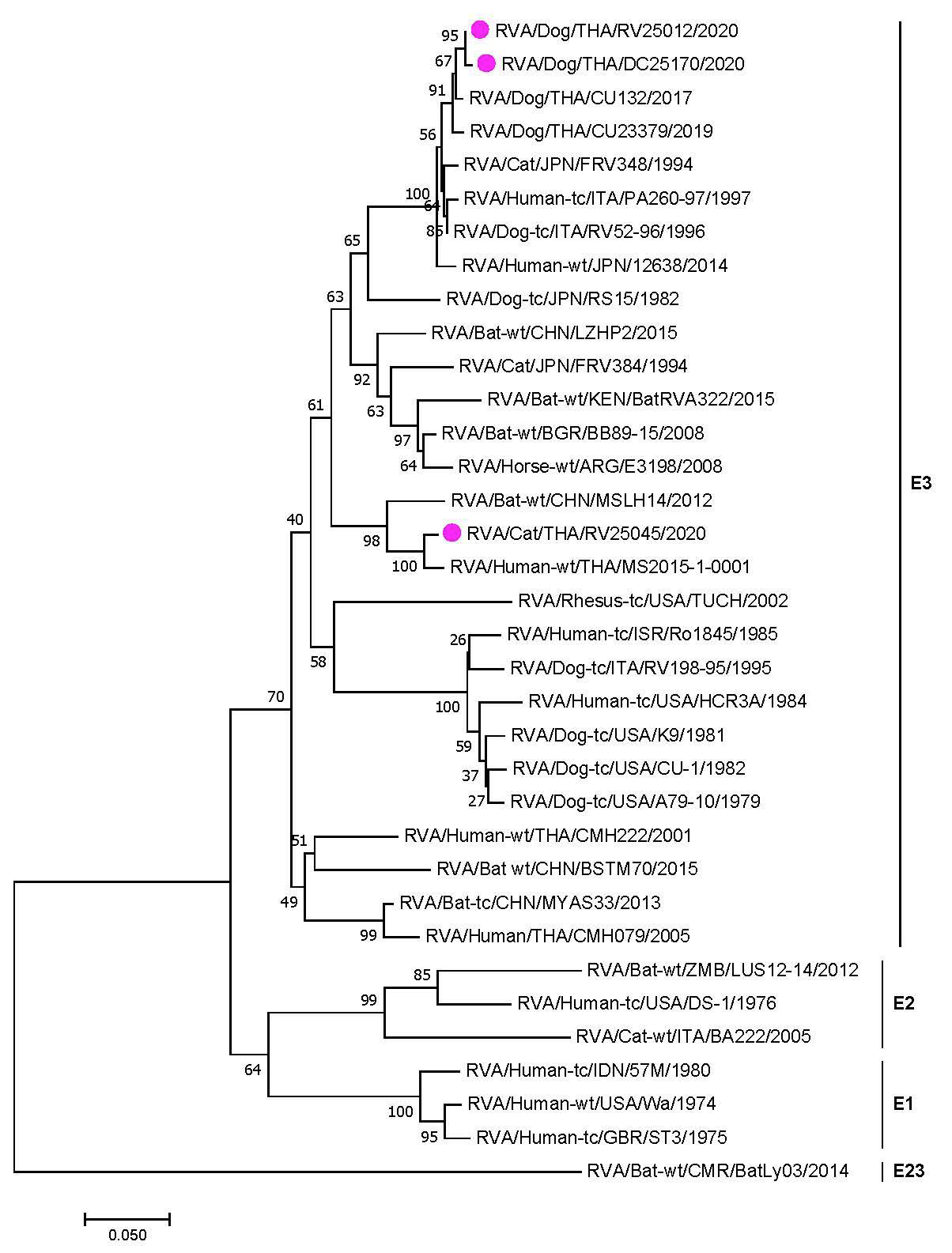


**NSP5**


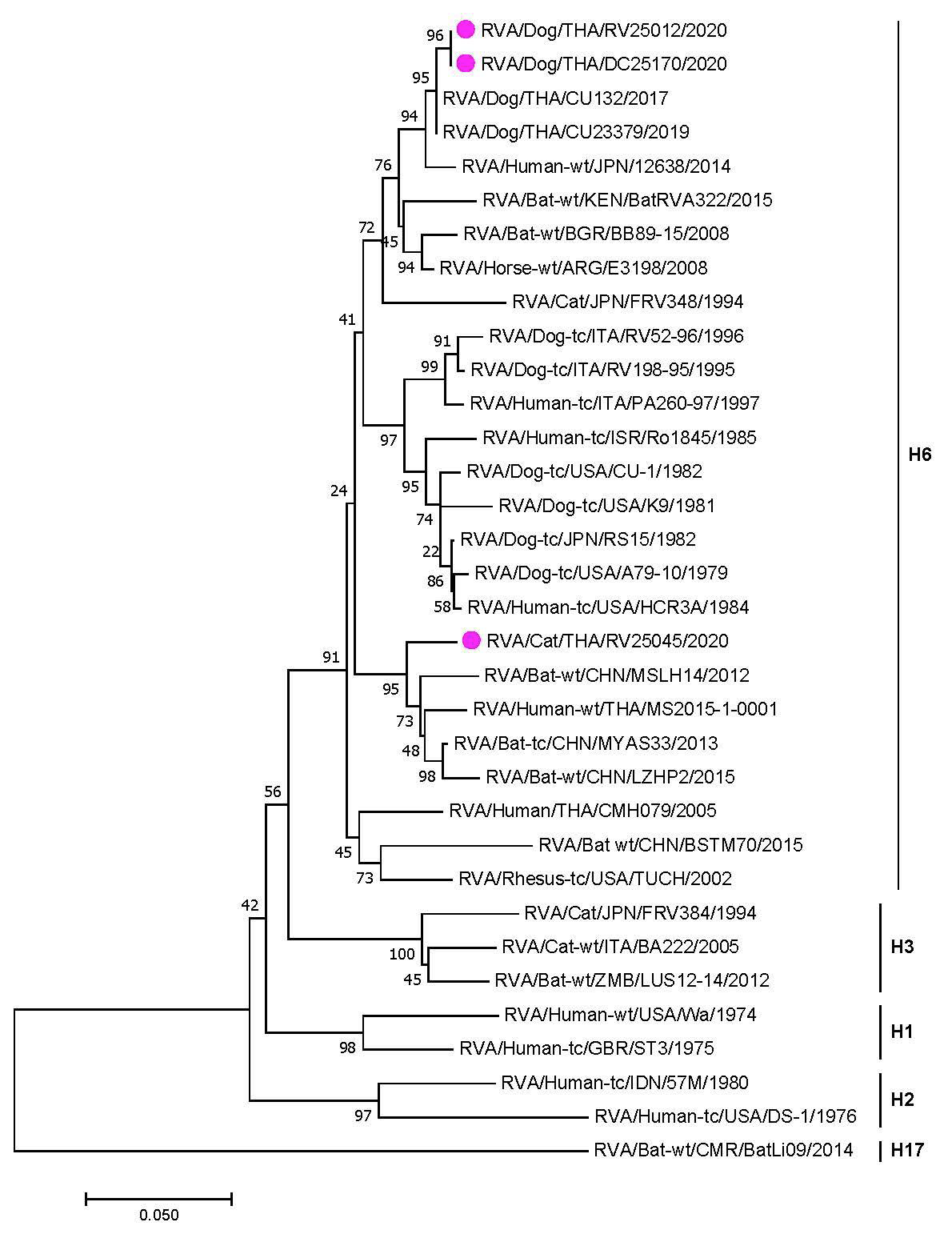

Supplement: Supplementary file 1 [file Data_Sheet_1.docx]
